# Genetic association of spikelet abortion with spike, grain, and shoot traits in highly-diverse six-rowed barley

**DOI:** 10.3389/fpls.2022.1015609

**Published:** 2022-11-21

**Authors:** Roop Kamal, Quddoos H. Muqaddasi, Thorsten Schnurbusch

**Affiliations:** ^1^ Leibniz Institute of Plant Genetics and Crop Plant Research (IPK), Gatersleben, Germany; ^2^ Faculty of Natural Sciences III, Institute of Agricultural and Nutritional Sciences, Martin Luther University Halle-Wittenberg, Halle, Germany

**Keywords:** final spikelet number, grain traits, grain morphometry traits, potential spikelet number, maximum yield potential, shoot traits, spike traits, spikelet abortion

## Abstract

Spikelet abortion is a phenomenon where apical spikelet primordia on an immature spike abort. Regardless of the row-type, both apical and basal spikelet abortion occurs, and their extent decides the number of grain-bearing spikelets retained on the spike—thus, affecting the yield potential of barley. Reducing spikelet abortion, therefore, represents an opportunity to increase barley yields. Here, we investigated the variation for apical spikelet abortion along with 16 major spike, shoot, and grain traits in a panel of 417 six-rowed spring barleys. Our analyses showed a significantly large genotypic variation resulting in high heritability estimates for all the traits. Spikelet abortion (SA) varies from 13 to 51% depending on the genotype and its geographical origin. Among the seven spike traits, SA was negatively correlated with final spikelet number, spike length and density, while positively with awn length. This positive correlation suggests a plausible role of the rapidly growing awns during the spikelet abortion process, especially after Waddington stage 5. In addition, SA also showed a moderate positive correlation with grain length, grain area and thousand-grain weight. Our hierarchical clustering revealed distinct genetic underpinning of grain traits from the spike and shoot traits. Trait associations showed a geographical bias whereby European accessions displayed higher SA and grain and shoot trait values, whereas the trend was opposite for the Asian accessions. To study the observed phenotypic variation of SA explained by 16 other individual traits, we applied linear, quadratic, and generalized additive regression models (GAM). Our analyses of SA revealed that the GAM generally performed superior in comparison to the other models. The genetic interactions among traits suggest novel breeding targets and easy-to-phenotype “proxy-traits” for high throughput on-field selection for grain yield, especially in early generations of barley breeding programs.

## Introduction

Increasing grain yield (GY) remains one of the major goals of barley (*Hordeum vulgare* L.) breeders and geneticists. GY is a complex trait mainly influenced by the concerted action of several agronomic/morphological traits such as plants per m^2^, spikes per m^2^, spikelets per spike, spike length, grain number per spike, grain weight, thousand-grain weight, and plant height. Therefore, it is of utmost importance to study the variation and relationship among these traits in order to better understand the contribution of these traits towards GY. One of the promising areas to improve barley GY is to increase the spikelet number per spike, which could be achieved by understanding the spikelet abortion process and its association with other spike, grain, and shoot traits.

During the early reproductive phase of a barley plant, the inflorescence meristem keeps producing spikelet primordia until the maximum yield potential (MYP) stage is reached; after this stage, the number of spikelet primordia plateaus (Thirulogachandar and Schnurbusch, 2021). The total spikelet primordia number obtainable at or after the MYP stage is known as the potential spikelet number (PSN) for a given immature spike. However, not all spikelet primordia survive until the grain filling phase. Due to apical spikelet abortion, spikelet primordia abort from the tip, thereby decreasing the actual harvestable GY of barley. This spikelet “initiation-and-degeneration” dynamics resuls in a proportion of the total spikelet primordia (Kirby and Appleyard, 1987; del Moral et al., 2003; González et al., 2003). Spikelet primordia that survive until heading and reach the grain filling phase are the final spikelet number (FSN). Since GY is also determined by the FSN harboring grains, it is crucial to study the causal relationship of spikelet abortion extent with FSN and other major agronomically important traits.

Previously, Alqudah and Schnurbusch (2014) showed that spikelet survival was highly genetically controlled as the heritability was ~0.80. Kamal et al. (2022) reported similar results, where the broad-sense heritability for percentage of spikelet abortion (SA%) was 0.81. Although, SA has high heritability that governs its genetic nature, it is still a plastic trait in barley as the number of aborted spikelets varies among the main culm and secondary tillers, genotypes and between row-types. Six-rowed barleys reported to display higher variation for the aborted spikelets as the lateral spikelets are fertile due to the loss of function of *Vrs1* gene (Miralles and Richards, 2000; Garcıa del Moral et al., 2002; Arisnabarreta and Miralles, 2004; Komatsuda et al., 2007; Alqudah and Schnurbusch, 2014). In addition, the extent of spikelet abortion is also affected by the ambient environmental conditions, e.g., temperature, seasons, and locations. It was speculated that lower temperatures between MYP stage and spike emergence stages lead to more survived spikelets (Ellis and Kirby, 1980). Survival of the spikelets was also reported to be different between the main culm and tillers where the former bear more spikelets than the latter (Cottrell et al., 1985).

Similar to spikelet abortion in barley, floret abortion occurs in wheat. Due to the indeterminate nature of the wheat spikelet, multiple florets are produced within each spikelet; however, only 3-4 florets survive and later develop into grains Therefore, the number of surviving fertile florets in wheat is also the result of the dynamic “floret initiation and abortion” process. Several practices, such as shortening the photoperiod and increasing the nitrogen fertilizer, improved the number of fertile florets during anthesis primarily by increasing floret survival (Reynolds et al., 2012; González-Navarro et al., 2015; Zhang et al., 2021). One of the hypotheses for floret or spikelet abortion is the competition between the stem and spike (Kirby, 1988; Arisnabarreta and Miralles, 2004). In wheat, numerous studies (Miralles et al., 1998; Gonzalez et al., 2011; Zhu et al., 2019; Zhang et al., 2021) focused on reducing the assimilates partitioning to the stem and diverting the assimilates to the spike. This is because spike dry matter weight is known to have a significant positive correlation with number of fertile florets, which, in turn, shows a positive shows a positive correlation with grain number per spike (GNS). Based on the available wheat literature, two models, namely the trophic model and the developmental model, were proposed to explain the mechanism governing number of fertile florets and grain numbers (Ferrante et al., 2013). The trophic model describes that floret death is triggered by the dynamics of the spike dry weight between the initiation of terminal spikelet and anthesis. On the other hand, the developmental model proposes that floret abortion is triggered by a fixed developmental stage of the most advanced floret primordium (Bancal, 2008; Bancal, 2009). This implies that the grain number has a stronger correlation with the time of floret death. Afterwards, Thirulogachandar et al. (2021) studied 27 barley accessions (17 two- and ten six-rowed barleys) to explain grain number determination and interactions between maximum spikelet number with other yield component traits and proposed two models, namely, the survival model and the developmental model. To understand the impact on grain number, they studied its association with PSN and spikelet survival. Their results showed that, in six-rowed barleys, GNS is associated with spikelet survival whereas, in two-rowed barley, GNS is strongly associated with PSN.

Since SA is highly laborious to phenotype and requires the tracking of the MYP stage in each accession under study, earlier studies in wheat and barley were confined to a few accessions. Here, we studied the variation for spikelet abortion in a diverse worldwide panel of 417 six-rowed barley accessions and its relationship with 16 other spike, grain and shoot traits. Previously, Kamal et al. (2022) explored the relationship between PSN, FSN, SA along with heading date and plant height. It was concluded that in six-rowed barley, FSN is mainly determined by the extent of SA and FSN and PSN together explained 93% of the variation for SA. They also reported that heading date does not directly affect the extent of SA; however, it indirectly affects SA by altering number of spikelet primordia formed during early reproductive stage. Nevertheless, apart from PSN and FSN, several other morphological traits such as spike length, spike weight, awn length, grain morphometric traits, grain set, thousand grain weight, and culm dry weight were not included in the previous studies. However, these traits could directly affect or interact with SA.

Here we present analyses of 16 spike, grain, and shoot traits to study (1) their genotypic variation, (2) their interaction, and (3) their trait associations with respect to the geographical origin—all in relation to SA. We report that among the spike traits, only awn length positively correlates with SA and that SA, in turn, influences grain morphometric traits—thus, representing a possible sink competition for grain yield. Our hierarchical clustering points to a distinct genetical underpinning of grain traits from spike traits. We have also analyzed the data with different regression methods to check other non-linear associations among the traits. Our results provide a deeper understanding of the genetic interaction between SA and the major spike, grain, and shoot traits.

## Material and methods

### Plant materials

A panel of 417 six-rowed spring barleys was evaluated at the fields of Leibniz Institute of Plant Genetics and Crop Plant Research, Gatersleben, Germany (51°49’23’’N, 11°17’13’’E, 112 m altitude) for three consecutive years (2018–2020). Each year, three replications were planted, and three main culms were selected and tagged in the center of each plot (replication) for data collection. All the accessions were selected based on their spring growth habit, *Ppd-H1* sensitive allele and accessions imitating the genetic diversity harbored in the Federal *ex-situ* German genebank. The comprehensive details for the panel selection criteria and field trails are described in Kamal et al. (2022). The majority of the panel consist of landraces (*n* = 350; 84%) along with a decent proportion of recent cultivars (*n* = 67; 16%). Standard agronomic practices were applied except for the plant growth regulators.

### Phenotyping

We studied 17 traits broadly divided into three categories, viz., spike (*n* = 7), grain (*n* = 7) and shoot (*n* = 3) traits (**Table 1**). In 2018, completely randomized design (CRD) was used, while in the years 2019 and 2020, the panel was grown in an α -lattice design. The individual plot size was ~1.5 m^2^, with each plot divided into six rows spaced 0.2 m apart and the sowing density was kept constant across the years with 20 kernels row^-1^. Comprehensive details of the experiment design are mentioned in Kamal et al. (2022). For PSN data collection, the MYP stage (Thirulogachandar and Schnurbusch, 2021) was tracked and three main culms per replication and accession (in total, nine spikes per accession per year) were selected for microscopic analyses. Not all the accessions reached the MYP stage simultaneously; therefore, main culms for each accession were collected on routine basis starting from the stem elongation phase (Anderson et al., 1995). Upon dissection under the stereomicroscope (Stemi 2000-c, Carl Zeiss Micro-Imaging, GmbH, Göttingen, Germany), individual rachis nodes (both differentiated and undifferentiated) were counted on the immature spikes. The total rachis node number was multiplied by three to obtain PSN. Later, after heading, three main culms were selected to calculate the number of spikelet per spike after the abortion process. The retained spikelets represented FSN. The same culms were later harvested and spike length (SL, cm), spike weight (SW, g), spike density (SW, g), spike density (SD calculated as ratio of final node number, i.e., FNN and SL) and awn length (AL) were measured. AL was measured on an ordinal scale ranging from 1–6 (**Figure S1**). As, spikelet abortion occurs over a few Waddington stages, it is difficult to track the spikelet abortion process in such a big panel. Therefore, we selected two developmental stages namely, MYP stage and heading date to calculate PSN and FSN, respectively. SA was calculated as


(1) $$\text{SA}\left(\%\right)=100-\left(\frac{\text{Final spikelet number }\left(\text{FSN}\right)}{\text{Potential spikelet number }\left(\text{PSN}\right)}\times 100\right)$$

**Table 1:** List of spike, grain and shoot traits evaluated on a panel of 417 six-rowed barley accessions collected from across the globe.

Sr. No.	Spike traits	Grain traits	Shoot traits
1	Potential spikelet number (PSN)	Grain number per spike (GNS)	Heading date (HD, days from 1^st^ January)
2	Final spikelet number (FSN)	Grain length (GL, mm)	Plant height (PH, cm)
3	Spikelet abortion* (SA, %)	Grain width (GWi, mm)	Culm dry weight (CDW, g)
4	Spike length (SL, cm)	Grain area (GA, mm^2^)	
5	Spike weight (SW, g)	Grain weight per spike (GWe, g)	
6	Spike density (SD)	Grain set (GS)	
7	Awn length (AL)	Thousand-grain weight (TGW, g)	

* In the text, the abbreviation “SA” represent the trait calculated from PSN and FSN using equation 1 whereas spikelet abortion represents the “in-between ongoing” abortion process during the early reproductive stages.

In the text, the abbreviation “SA” represent the trait calculated from PSN and FSN using equation 1 whereas spikelet abortion represents the “in-between ongoing” abortion process during the early reproductive stages. The grain traits namely grain number spike (GNS), grain length (GL, mm), grain width (GWi, mm), grain area (GA, mm^2^), grain weight per spike (GWe, g) and thousand-grain weight (TGW, g) were measured using a digital seed analyzer “Marvin” (GTA Sensorik GmBH, Neubrandenburg, Germany). Marvin analyzer takes into account GNS to calculate TGW as [(GWe/GNS) × 1000]. Lastly, grain-set (GS; %) was calculated from the ratio of GNS to FSN. Among the shoot traits, heading date (HD) was calculated as the number of days from January 1^st^ until 50% of the spikes were out from the flag leaf sheath. Plant height (PH, cm) was measured on the same three main culm selected earlier for FSN calculation as a distance from the soil surface to the base of the spike and culm dry weight (CDW, g) was measured after harvest.

### Variance component analyses and calculation of BLUEs

We employed two field designs, namely, CRD in 2018 and α-lattice in 2019 and 2020. The field design was changed from CRD to α-lattice as the latter is known to capture the genetic effects more accurately. Consequently, two different linear mixed-effect models were used to compute individual variance components. In 2018, within-year data analysis was performed by assuming all effects except the intercept as random in eq. 2:


(2) $$y_{ij}=\mu +g_{i}+r_{j}+\epsilon_{ij}\;$$

where, *y_ij_* is the phenotypic record of the *i^th^* genotype in *j^th^* replication, *µ* is the common intercept term, *g_i_* is the effect of the *i^th^* genotype, *r_j_* is the effect of the *j^th^* replication and *ε_ij_* denotes the corresponding residual term.

For 2019 and 2020 within-year data analyses, we used the eq. 3 by assuming all effects except the intercept as random as:


(3) $$y_{ijk}=\mu +g_{i}+r_{j}+\beta_{\left(j|k\right)}+\epsilon_{ijk}\;$$

where, *y_ijk_* is the phenotypic record of the *i^th^* genotype in the *j^th^* replication and *k^th^* block, *µ* is the common intercept term, *g_i_* is the effect of *i^th^* genotype, *r_j_* is the effect of the *j^th^* replication, *β*_(_ *_j|k_*_)_ is the block effect of the *k^th^* block nested in the *j* ^*th*^ replication and *ε_ijk_* is the corresponding residual term. Within-year repeatability (*Ĥ* ^2^) was calculated as:


(4) $${\hat{H}}^{2}=\frac{\sigma_{g}^{2}}{\sigma_{g}^{2}+\left(\frac{\sigma_{\epsilon }^{2}}{n_{R}}\right)}$$

where, *σ* ^2^_g_ and *σ* ^2^ *_ε_* represent the genotypic and residual variances, respectively; *n_R_* denotes the within-year number of replications.

Except for SA, SD, and GS, the across-years variance component analyses were performed by assuming all effects except the intercept as random in eq. 5 as:


(5) $$y_{ijkl}=\mu +g_{i}+y_{j}+\;{\left(g\times y\right)}_{\left(ij\right)}+\;{\left(y\times r\times \;\beta \right)}_{\left(j\left|k\right|l\right)}+\;\epsilon_{ijkl}\;$$

where, *y_ijk_* is the phenotypic record of the *i^th^* genotype in the *j^th^* year and *k^th^* replication nested in *l^th^* block, *µ* is the common intercept term, *g_i_* is the effect of *i* ^*th*^ genotype, *y_j_* is the effect of the *j^th^* year, (g×*y*)_(_ *_ij_*_)_ is the genotype-by-year interaction effect of the *i^th^* genotype and *j^th^* year, (*y*×*r*×*β*)_(_ *_j_*_|_ *_k_*_|_ *_l_*_)_ is the *l^th^* block nested in *k* ^*th*^ replication in *j^th^* year, and *ε_ijkl_* is the corresponding residual term. The across-years broad-sense heritability (*H* ^2^) was calculated as:


(6) $$H^{2}=\frac{\sigma_{g}^{2}}{\sigma_{g}^{2}+\left(\frac{\sigma_{g\times y}^{2}}{n_{y}}\right)+\left(\frac{\sigma_{\epsilon }^{2}}{n_{y}\times n_{R}}\right)}$$

*w*here *σ* ^2^_g_, *σ* ^2^_g×_ *_y_*, and *σ* ^2^ *_ε_* denote the genotypic, genotype-by-year, and the residual variance, respectively; *n_y_* and *n_R_* represent the average number of years and number of replications, respectively.

Since SA, SD and GS were derived traits, we used the following model to compute the variance components of genotype and years:


(7) $$y_{ij}=\mu +g_{i}+y_{j}+\epsilon_{ij}$$

where, *y_ij_* is the phenotypic record of the *i^th^* genotype in the *j^th^* year, *µ* is the common intercept term, *g_i_* is the effect of the *i^th^* genotype, *y_j_* is the effect of the *j^th^* year and *ε_ij_* is the corresponding residual term. Accordingly, the *H* ^2^ was calculated based on eq.4, except that *n_R_* is replaced with *n_y_*.

### Principal component and correlation analyses

We drew a scree-plot to describe the percentage of variation accounted for by each principal component (PC) and the principal component analysis (PCA) plot to explain the relationships among the traits. A projection from the origin represented each trait. The length of the projection of a given trait from its origin measured the quality of the trait on the plot. In PCA plot, traits that are away from the origin are the major contributors of the corresponding PC. We also calculated the major trait contributors for the first five PCs.

Pearson’s product-moment correlation (*r*) was computed to examine the relationship among the traits. Moreover, to check the across-years general performance of a given trait, we computed average correlation $\left(\bar{r}\right)$ by performing Fisher’s *z* transformation, as described in (Muqaddasi et al., 2020). Since the panel consists of accessions collected from five different continents, we used the student *t*-test to check if significant (*P<*0.05) differences exist for a trait with respect to geographical origin of the accessions.

### Regression analyses: linear, quadratic, and generalized additive models

We implemented and compared linear (Su et al., 2012), quadratic (Bobbitt, 2020) and generalized additive model (GAM) (Wood, 2006) regression analyses to check the relationship between SA and all other traits. For the linear regression, the following model was used:


(8) $$y=\;\beta_{0}+\;\beta_{p}x_{p}+\epsilon \;$$

where *y* is the response trait i.e., SA, *β*_0_ is the intercept, *β_p_* is the regression coefficient, and *x_p_* is a given predictor trait. To check the quadratic relationship among the traits, we used the following model:


(9) $$y=\beta_{0}+\beta_{1}x_{p}+\beta_{2}x_{p}^{2}+\epsilon \;$$

where *y* is the response trait, *β*_0_ is the intercept, *β*_1,_ *β*_2_ are the regression coefficients, *x_p_* is the predictor trait, and $x_{p}^{2}$ is the quadratic function of that trait. In situations where linear and quadratic regression failed to explain the relationship between the traits, we used the generalized additive model (GAM) as follows:


(10) $$y=\beta_{0}+s_{1}(x_{1})+s_{2}\left(x_{2}\right)+\;.\;.\;.\;+s_{p}(x_{p})+\epsilon \;$$

where *y* is the response trait, *β*_0_ is the intercept, *s*_1_, *s*_2_,…, *s*_*p*_ are the smooth function in GAM, and *x*_1_, *x*_2_,…, *x*_*p*_ are the predictor traits. Since GAMs combine the generalized linear model and additive model, they are not restricted to the normal distributions. Instead, these models used smoothening (splines or LOESS) functions that separate the data into “smooth + rough” parts to maximize the smooth and minimize the rough part. In addition to implementing different models, we performed ANOVA to examine the model fit, i.e., to check which model significantly better capture the variation in SA with respect to a given predictor trait. Unless stated otherwise, all calculations were performed in R software (Team, R.C, 2013) using the packages lme4 (Bates et al., 2015), corrplot, factoextra, factoMineR (Lê et al., 2008) and mgcv (Wood and Wood, 2015).

## Results

### Variance component analyses show large genotypic variance and high heritability estimates

We evaluated 417 six-rowed spring barleys for variation in seven spike-related traits (PNS, FSN, SA, SL, SW, SD, and AL), seven grain traits (GNS, GL, GWi, GA, GWe, GS, and TGW) and three shoot (HD, PH, and CDW) traits to study the relationship between three classes of morphological traits. Restricted maximum likelihood based variance component analyses showed that both within- and across-years genotypic variance was significantly (*P*<0.001) greater than zero and was the principal contributor for variance in these traits (**Tables S1**−**S4**).

**Figure 1:**
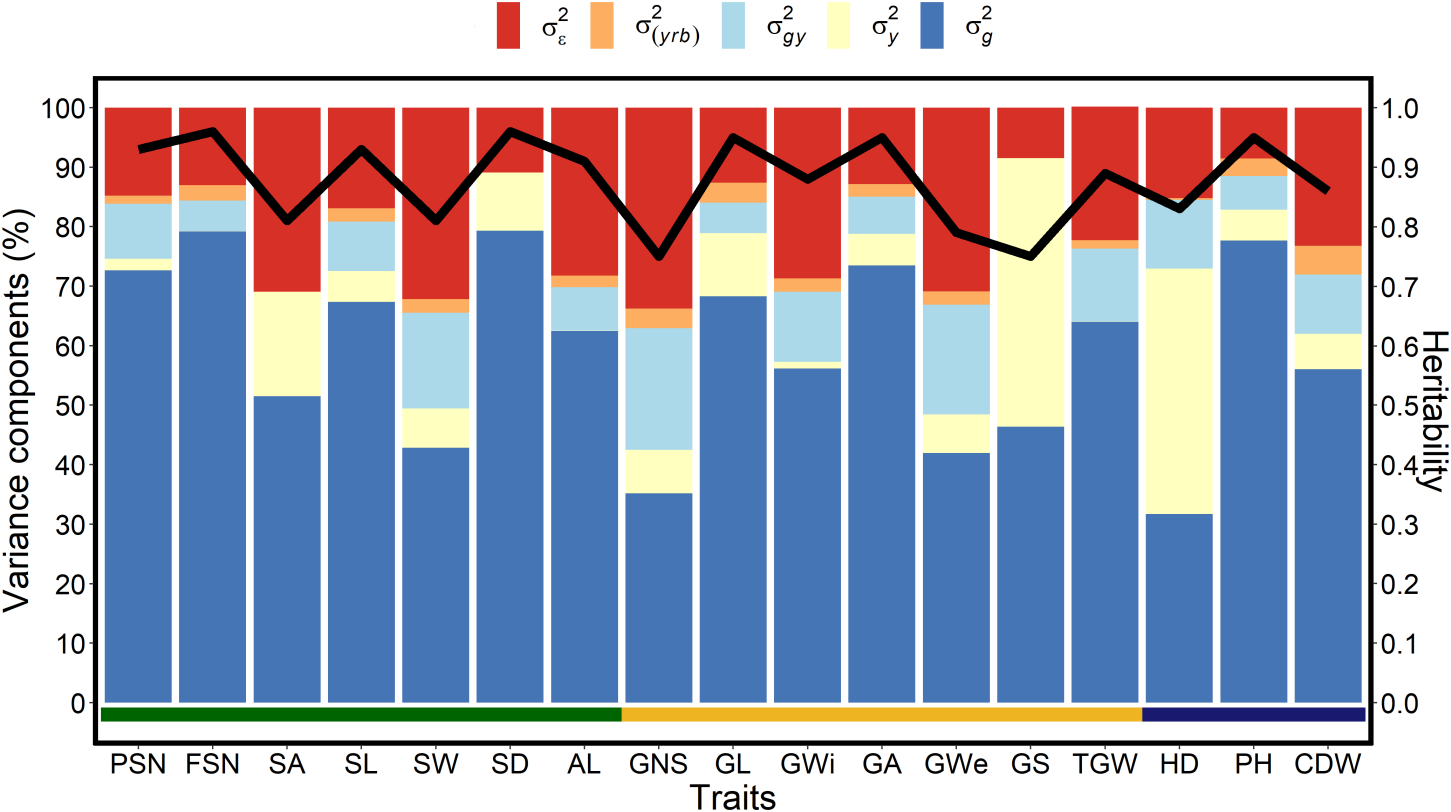
Proportion of the different variance components and heritability for each investigated trait in a panel of 417 six-rowed spring barley accessions. The *x*-axis represents all investigated traits, the left *y*-axis denotes the proportion of the variance components in percent, and the right *y*-axis represents the heritability scores. The black line represents the heritability value for the respective trait, $\sigma_{g}^{2}$ is the genotypic variance, $\sigma_{y}^{2}$ is the year variance, *σ* ^2^_g_ *_y_* is the (genotype × year) interaction variance, *σ* ^2^_(_ *_yrb_*_)_ is the year and replication variance with replication nested into the blocks and $\sigma_{\epsilon }^{2}$ is the error or residual variance. The spike traits indicated by the green horizontal line includes potential spikelet number (PSN), final spikelet number (FSN), spikelet abortion (SA in %), spike length (SL in cm), spike weight (SW in g), spike density (SD) and awn length (AL). Grain traits represented by the yellow horizontal line includes grain number per spike (GNS), grain length (GL in mm), grain width (GWi in mm), grain area (GA in mm^2^), grain weight per spike (GWe in g), grain set (GS in %), thousand-grain weight (TGW in g) and the shoot traits represented by blue horizontal line includes heading date (HD in days from January 1^st^), plant height (PH in cm) and culm dry weight (CDW in g).

The genotypic variance trend for spike traits was also reflected in broad-sense heritability (*H* ^2^) estimates with highest value for SD (*H* ^2^ = 0.96) and lowest for SW (*H* ^2^ = 0.81) (**Figure 1**). Among grain traits, GNS, GWe and GS showed phenotypic plasticity either because of greater year variance or residual variance, whereas, GA, GL, and TGW were generally stable. The high genotypic variance for GA and GL resulted in the highest heritability estimates for both the traits (*H* ^2^ = 0.95), while the least heritability was observed for GNS (*H* ^2^ = 0.75) (**Figure 1**). For shoot traits, across-year analyses showed that PH possess the highest genotypic variance, followed by CDW and HD (**Figure 1**). Because of temperature fluctuations and slightly different sowing dates over three years, we observed a higher yearly effect for HD. Nonetheless, the large genotypic variance also resulted in high heritability estimates for all the shoot traits (**Figures 1**, **S2**).

**Figure 2:**
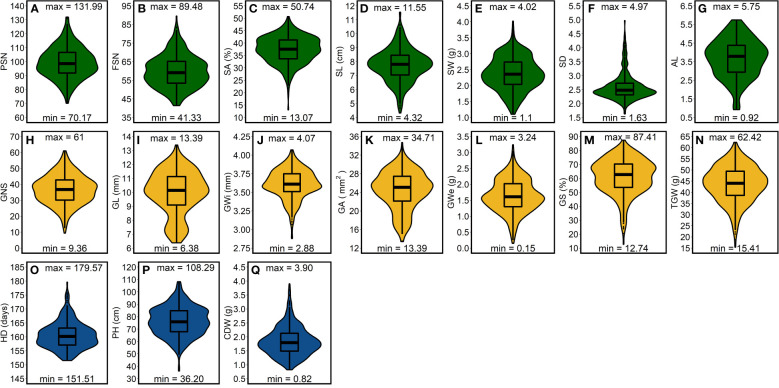
Phenotypic distribution of the investigated traits in a panel of 417 six-rowed spring barley accessions. **(A-G)** frequency distribution for the spike traits, **(H-N)** frequency distribution for grain traits and **(O-Q)** frequency distribution for shoot traits. “max” and “min” represents the maximum and minimum value for each investigated trait and the box plot within the violin plots represents the lower quartile, median and upper quartile for each trait. PSN = potential spikelet number; FSN = final spikelet number; SA = spikelet abortion (SA in %); SL = spike length (in cm); SW = spike weight (in g); SD = spike density; AL = awn length; GNS = grain number per spike; GL = grain length (in mm); GWi = grain width (in mm); GA = grain area (in mm^2^); GWe = grain weight per spike (in g); GS = grain set (in %); TGW = thousand-grain weight (in g); HD = heading date (in days from January 1^st^); PH = plant height (in cm) and CDW = culm dry weight (in g).

The best linear unbiased estimations (BLUEs)—calculated within- and across-years—exhibited large genotypic variation (**Figures 2**, **S3–5**). Among spike traits, average correlations—calculated to observe the consistency of the phenotypic data across three growing years—was highest for SD $\left(\bar{r}=0.89\right)$ and lowest but reasonably good for SA $\;(\bar{r}=0.61$ ; **Figures S6A–G**). In general, average correlation was high for all the spike traits, suggesting the suitability of the data to draw accurate conclusions. Furthermore, we also observed high average correlation values for all the grain traits with highest for GL ( $\bar{r}=0.91$ ; **Figures S6H–N**). The average correlation $\left(\bar{r}\right)$ amounted to 0.59 for HD, 0.80 for CDW and 0.92 for PH (**Figures S6O–Q**)—this reveals a relatively more significant environmental impact on HD than PH and CDW.

### Principal component analysis revealed the opposite genetic nature of spike and grain morphological traits

**Figure 3:**
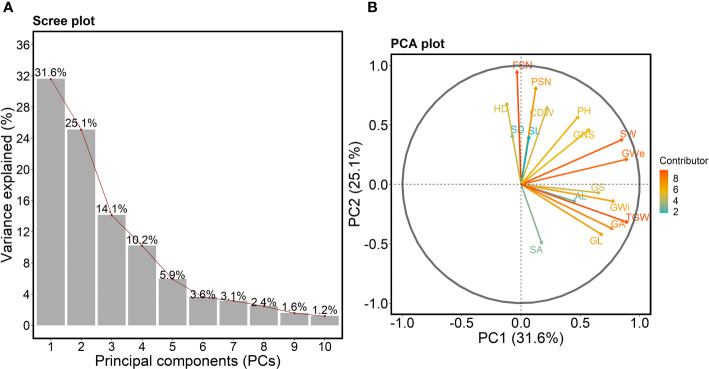
**(A)** Scree plot highlighting the first ten principal components (PCs) describing the total variation present for all the studied traits. The *x-* and *y-*axis represent the first 10 PCs and variance explained in percent, respectively. The red line shows the trend of the percentage of variance explained starting from PC1 to PC10. **(B)** PCA plot showing the PC1 on the *x*-axis and PC2 on the *y-*axis. The contribution of each trait towards PC1 and PC2 is indicated by the color-coded arrow with maximum contributors indicated by orange to red arrows and minimum contributors by cyan-colored arrows. The legend describes the strength of the contribution. PSN = potential spikelet number; FSN = final spikelet number; SA = spikelet abortion (SA in %); SL = spike length (in cm); SW = spike weight (in g); SD = spike density; AL = awn length; GNS = grain number per spike; GL = grain length (in mm); GWi = grain width (in mm); GA = grain area (in mm^2^); GWe = grain weight per spike (in g); GS = grain set (in %); TGW = thousand-grain weight (in g); HD = heading date (in days from January 1^st^); PH = plant height (in cm) and CDW = culm dry weight (in g).

We performed principal component analysis (PCA) on all 17 traits to check the variation and major contributing traits for first five PCs. The scree-plot showed that first five PCs together explained 86.7% of the total variation in the data set (**Figure 3A**). Interestingly, PC_1_, explaining 31.6% variance, showed that all the grain traits and SW vary together (**Figures 3B**, **S7**). However, PSN, FSN and shoot traits act differently—with all being in PC_2_.

In PCA plot (**Figure 3B**), the angle between the arrows/projections illustrates the relationship between the traits: an acute angle depicts a positive association, a 90° angle shows no association, and an obtuse angle describes a negative association. Likewise, arrow length for a given trait explains its impact on a particular PC. For instance, the arrow length for TGW and FSN was longer than other traits indicating that TGW and FSN were the major contributors for PC_1_ and PC_2_, respectively (**Figure 3B**). The acute angle between SA and grain traits such as GL, GWi, GA, GS, TGW and one spike trait AL depicted a positive association among these traits.

We observed acute angles between shoot traits, PSN and FSN, suggesting alterations in HD directly affects the spikelet number and plant biomass accumulation. Similarly, obtuse angles between SA and most of the spike and shoot traits point towards the opposite nature of these traits, i.e., alternations in HD and consequently in PSN affects FSN, SL and SD further leading to changes in SA. We also checked the contribution of each trait towards the first five PCs (**Figure S7**). As mentioned earlier, the first five PCs were selected as they explained most of the total variation. TGW, FSN, GS, SD and SA were the major contributors and FSN, GS, FSN, GL and SW were the least contributors for PC_1_, PC_2_, PC_3_, PC_4_, and PC_5_, respectively (**Figure S7**). It is worth mentioning that the contribution to PC_5_ highlights the association between SA and AL—both the traits tend to vary together.

### Correlation analyses show stronger interdependencies among the grain traits as compared to spike and shoot traits

We performed the Pearson’s product-moment correlation (*r*) on within- and across-years BLUEs and observed a strongest positive association between PSN and FSN (**Figure S8A**), i.e., higher PSN leads to higher FSN. Both PSN and FSN were positively correlated with SL, SW and SD. The analysis showed that PSN was insignificantly correlated with SA whereas FSN showed a negative correlation with SA. Across-year analyses showed a negative correlation of SA with SD, i.e., denser spikes show more SA (*r* = –0.32; *P<*0.001); SA was, however, positively correlated with AL (*r* = 0.34; *P<*0.001; **Figure S8A**). As expected, SL and SD showed a negative correlation. We also observed inconsistent significance levels between year 2018 and the remaining years especially for the correlation between SA and PSN, SL, and AL. This could be attributed to the harsh weather condition in 2018 affecting the overall plant growth and the number of spikelet primordia produced on an immature spike.

In contrary to the spike traits, higher correlation coefficients were observed among the grain traits (**Figure S8B**). A high positive correlation was noticed among GNS, GWe, and GS (*r* >0.8). Usually, with an increase in GNS, TGW tends to decreases; however, a positive correlation was observed between GNS and TGW in both within- and across-year data analyses. GA showed higher correlation values with GL (*r* = 0.96) than GWi (*r* = 0.76). But, this trend was reversed for TGW, where GWi (*r* = 0.84) showed a higher correlation with TGW than GL (*r* = 0.78). Except for GA, GWi showed higher correlation coefficients for GNS, GWe, GS and TGW—as compared to GL—indicating that GWi could be an important factor deciding the grain parameters in barley. For shoot traits, HD was more significantly correlated with CDW than PH. PH and CDW also show a positive and significant correlation (**Figure S8C**).

### Hierarchical clustering highlights six distinct clusters separating spike and shoot traits from grain traits

**Figure 4:**
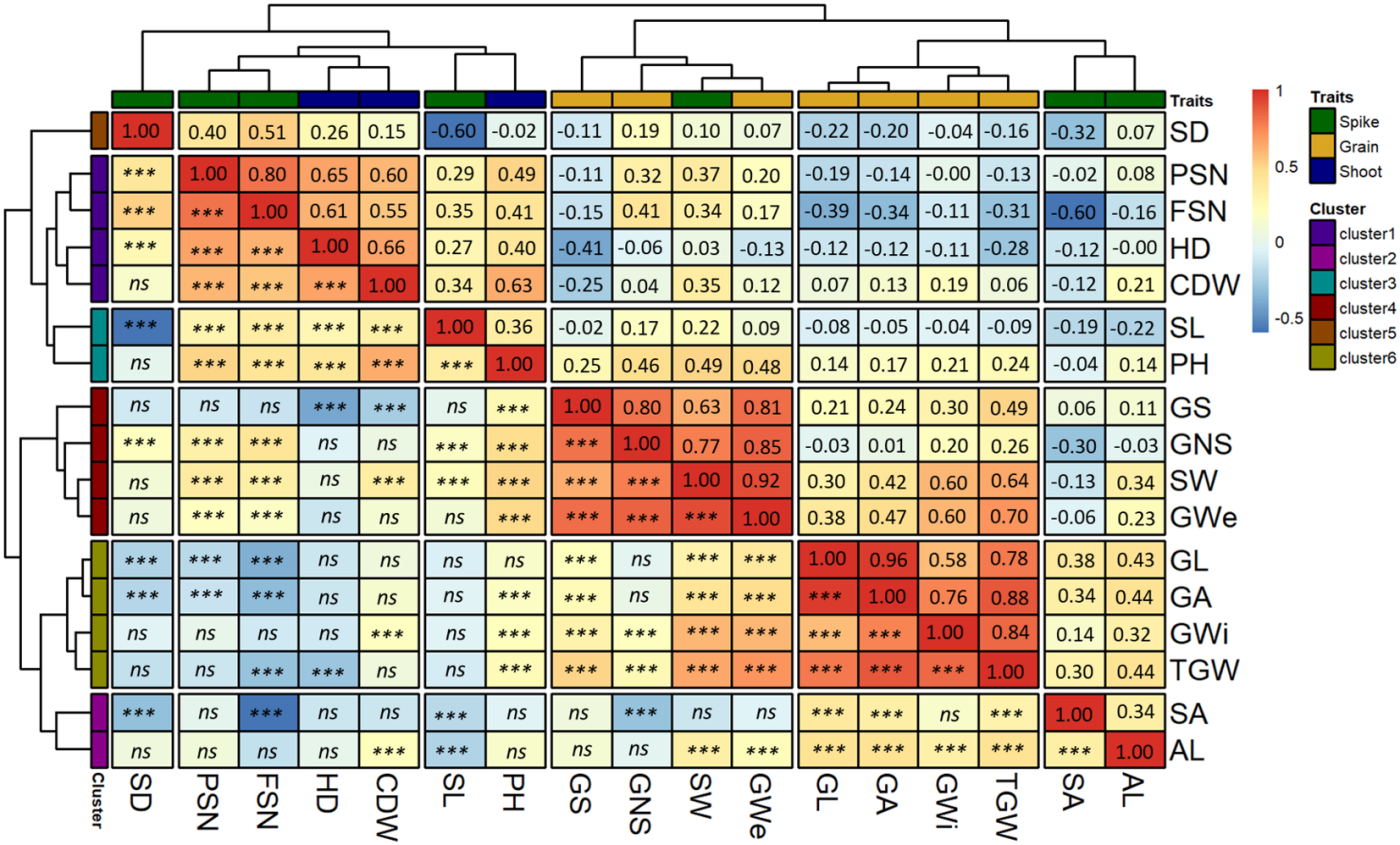
Hierarchical clustering based on the Pearson-product moment correlation analysis among the BLUEs of the investigated traits. The *x-* and *y-*axis both represent the investigated traits. The spike, grain and shoot traits are color-coded as shown in the legend “Traits”. The second legend shows the scale for Pearson-product moment correlation. The spike traits highlighted by the green color includes potential spikelet number (PSN), final spikelet number (FSN), spikelet abortion (SA in %), spike length (SL in cm), spike weight (SW in g), spike density (SD) and awn length (AL). Grain traits highlighted by the yellow color includes grain number per spike (GNS), grain length (GL in mm), grain width (GWi in mm), grain area (GA in mm^2^), grain weight per spike (GWe in g), grain set (GS in %), thousand-grain weight (TGW in g) and the shoot traits represented by blue color includes in heading date (HD in days from January 1^st^), plant height (PH in cm) and culm dry weight (CDW in g). *** = significant association at 0.001 probability level and ns represents non-significant association.

We performed hierarchical clustering based on the correlation matrix that divided all the traits into two main clusters. Broadly, grain traits were entirely separated from shoot traits, whereas spike traits were clustered with both grain and shoot traits (**Figure 4**). Further dissection of these two main clusters revealed six distinct sub-clusters.

Cluster-1 contained two shoot (HD and CDW) and two spikes (PSN and FSN) traits. Both HD and CDW were positively correlated with PSN and FSN suggesting the positive influence of HD and CDW on the spikelet primordia initiation. Across sub-clusters, CDW was positively correlated with all the spike traits except SA. In contrast, HD showed an insignificant correlation with SA, AL and SW, suggesting that spike characteristics and eventually grain number varies with both variation in days to heading and culm biomass.

Two spike traits, AL and SA were in cluster 2. Interestingly, AL was the only trait that showed a positive correlation with SA (*r* = 0.34) suggesting a role for awn growth during spikelet abortion process (further explained in the discussion section). Across clusters, all grain traits except GNS and GS also showed a positive correlation with AL. Hence, it could be speculated that simultaneous awn development and spikelet primordia growth during juvenile spike growth increases the number of aborted spikelets leading to lesser but bigger grains on the spike. 

Cluster-3 also harbored two traits, namely, SL and PH. The hierarchical clustering highlighted a higher association of these traits with other shoot and spike traits than grain traits. Cluster-4 composed of GNS, GWe, GS and one spike trait, SW. The highest correlation was observed between GWe and SW (*r* = 0.92). Furthermore, GNS and GS showed a positive correlation with SW, suggesting spikes with more fertile grains are heavier and wider—an important indirect selection criterion in high-throughput on-field phenotyping, especially early generations of breeding programs. We observed an insignificant correlation for GS with most of the spike traits except FSN and SW. Cluster-5 contained only SD which was closely placed with other spike and shoot traits. SD was positively related to GNS and HD but negatively with SA, GL, and GA.

Grain morphometric traits (GL, GWi and GA) and TGW were in cluster 6 (**Figure 4**), highlighting the close association of underlying genetic mechanisms for the grain morphometric traits with TGW. Both GL and GA were negatively correlated with PSN and FSN indicating that higher PSN or FSN leads to smaller grains. Similarly, TGW showed a negative correlation with FSN, i.e., higher the spikelet number after abortion, lower the TGW. Except PSN and FSN, cluster-6 traits positively correlated with SA, SW and AL. Therefore, from the positive correlation of grain traits, especially with SA, it can be concluded that an increase in SA leads to fewer spikelets and hence larger GL, GA and TGW. This happens through proper development of the grains that further increases SW.

### Geographical origin of the accessions significantly impact the variation for all the traits

**Figure 5:**
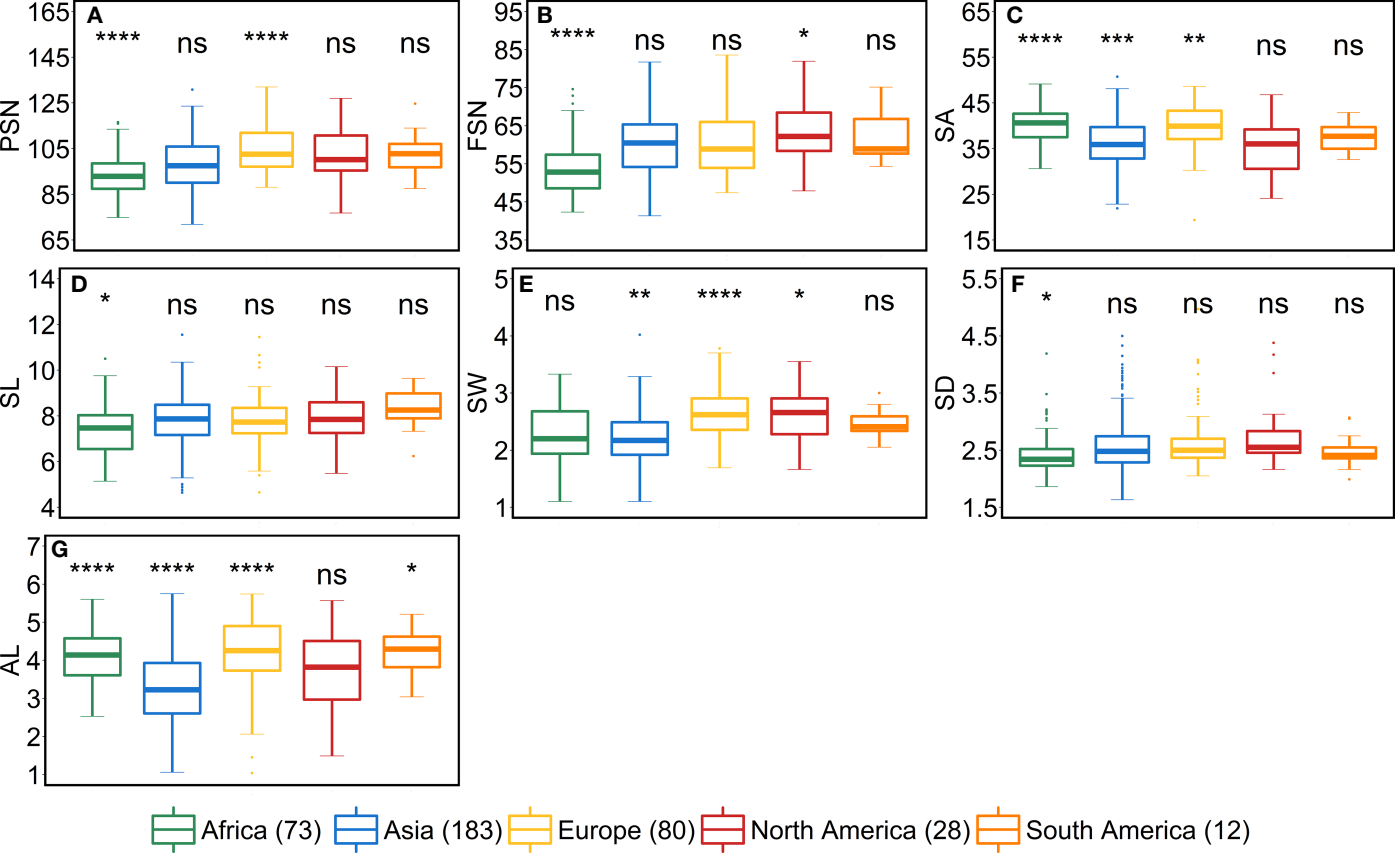
Comparison of accessions according to their geographical origin for the spike traits. **(A)** potential spikelet number (PSN), **(B)** final spikelet number (FSN), **(C)** spikelet abortion (SA in %), **(D)** spike length (SL in cm), **(E)** spike weight (SW in g), **(F)** spike density (SD) and **(G)** awn length (AL). ‘*n*’ denotes the number of accessions in this panel and the number of the accessions belonging to each continent; ****, ***, **, and * = significance level at the 0.0001, 0.001, 0.01, and 0.05 probability level, respectively and ‘ns’ represents insignificant differences and the legend represents different continents.

**Figure 6:**
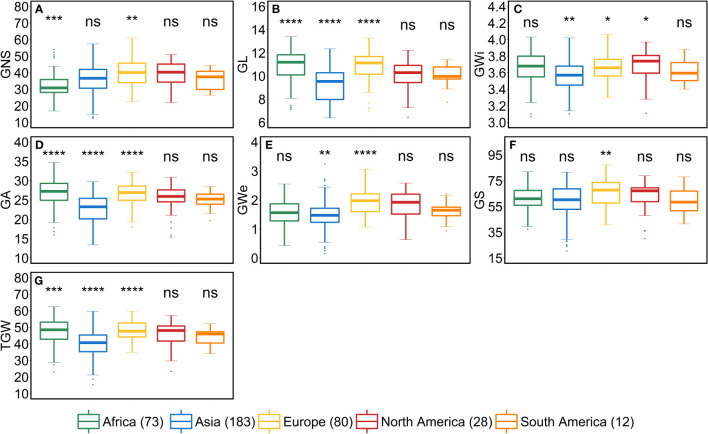
Comparison of accessions according to their geographical origin for the grain traits. **(A)** grain number per spike (GNS), **(B)** grain length (GL in mm), **(C)** grain width (GWi in mm), **(D)** grain area (GA in mm^2^), **(E)** grain weight per spike (GWe in g), **(F)** grain set (GS in %) and **(G)** thousand-grain weight (TGW in g). ‘*n*’ denotes the number of accessions in this panel and the number of the accessions belonging to each continent; ****, ***, **, and * = significance level at the 0.0001, 0.001, 0.01, and 0.05 probability level, respectively and ‘ns’ represents insignificant differences and the legend represents different continents.

**Figure 7:**
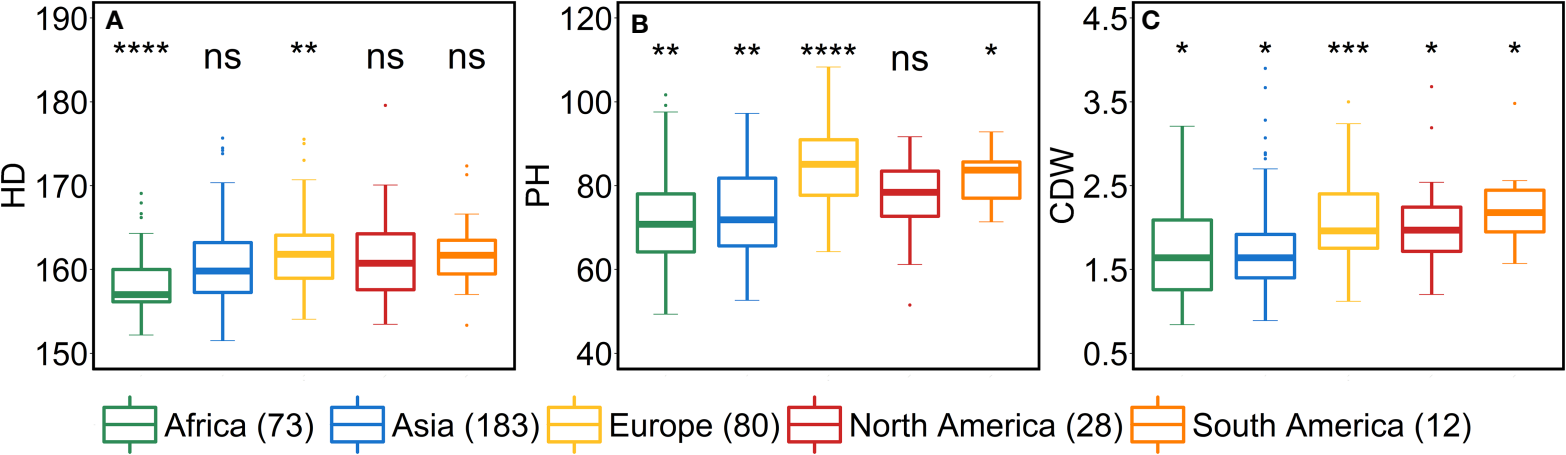
Comparison of accessions according to their geographical origin for the shoot traits. **(A)** heading date (HD in days from January 1^st^), **(B)** plant height (PH in cm) and **(C)** culm dry weight (CDW in g). ‘*n*’ denotes the number of accessions in this panel and the number of the accessions belonging to each continent; ****, ***, **, and * = significance level at the 0.0001, 0.001, 0.01, and 0.05 probability level, respectively and ‘ns’ represents insignificant differences and the legend represents different continents.

We studied the influence of geographical origin of the accessions on each trait by dividing the whole panel into six groups according to the continents, viz., African (*n* = 73), Asian (*n* = 183), European (*n* = 80), North American (*n* = 28), South American (*n* = 12) and accessions with unknown origin (*n* = 41). For comparison, we excluded the accessions with an unknown origin. For most of the traits, North- and South-American accessions did not show any significant differences from other continents (**Figures 5**–**7**). One reason for less variability within both the American accessions could be the low sample number as compared to other continents.

African accessions showed significant (*P*<0.001) lower values for PSN, FSN, SL, SD, GNS and HD. In nutshell, African accessions, took fewer days to head; therefore, immature spikes stay in spikelet initiation phase for a shorter period leading to lower PSN, consequently lower FSN, and ultimately lower GNS at harvest. Contradictorily, European accessions displayed higher values for all the shoot traits (**Figure 7**). As the HD increases in European accessions, spikelet initiation phase is also expected to be longer (higher PSN; **Figure 5**).

As stated above, a positive correlation was observed between SA and AL. We observed the same trend for African, Asian and European accessions. Asian accessions with lower AL showed lower SA, whereas, African and European accessions with higher AL have high SA. This further points to a potential role of awn development in affecting the extent of SA. However, the lower SA in Asian accessions leads to smaller grains—all grain morphometric traits and TGW were reduced (**Figure 6**). One possibility that could be exploited under European conditions is to pyramid the lower SA alleles from Asian accessions and higher grain size alleles from the European accessions in crosses and further test the relationship between the SA and grain size. Since accessions belonging to these continents can also be differentiated as landraces (*n* = 350) and cultivars (*n =* 67), we performed an analysis to check whether there exist any differences between landraces and cultivars for all the investigated traits. The significant differences were observed only for FSN, SA, SW and GNS. SA was higher for landraces than cultivars resulting in lower FSN and consequently lower GNS and SW (**Figure S9**). This hints towards the breeding progress for higher grain number and GY and consequently lower SA.

## Discussion

Early and late reproductive phases govern important agronomic traits in cereals. From a practical standpoint, early vigor and later stand strength correspond to better GY in barley and wheat. In early generations (e.g., F_4_, F_5_, DH1), breeders select the genotypes or plots based on the plant and spike ideotypes to further test in yield plots. Since GY is an integral parameter of many component traits, such as number of spikes per unit area, number of spikelets per spike, number of grain per spike, spikelet fertility, spike density, spike length, grain weight per spike and many more, the extent of SA directly or indirectly affect these traits. It is thus vital to study spikelet abortion from early to late reproductive stages and its association with other agronomically important traits.

### Plausible relationship between awn length, spikelet abortion, and grain development

Spikelet abortion is a laborious trait to phenotype as it entails the phenotyping of a given accession at both early (for PSN) and late reproductive (for FSN) stages. Previously, in barley, the relationship of SA with other traits was elucidated but in only a handful of genotypes. This is the first study where we report the genotypic association of SA with other spike, shoot, and grain traits at a large scale. As mentioned in the result section, AL was the only spike trait positively (moderately) correlated with SA. Depending on the growing conditions and genetic background of an accession, awns act as a boon and bane for a plant. (Vervelde, 1953; McKenzie, 1972; Knott, 1986; Weyhrich et al., 1994; Motzo and Giunta, 2002; Martin et al., 2003; Ali et al., 2010; Guo and Schnurbusch, 2016). The positive effect of the awns has been attributed to their role in photosynthesis i.e., awns can function as an important source organ because of their short nutrient transport route to grains, especially when flag leaves start to senesce (Li et al., 2006). While, the negative effect of awns arises as they act as a significant sink competitor of the growing spikelets for the available assimilates in an immature spike. Guo and Schnurbusch (2016) hypothesized that GY is influenced by the redistribution of assimilates within the spike associated with vigorous awn development. In wheat, it has been shown that the growing awns need more assimilates for their development as they represent 40% of the total spikelet biomass prior to spike emergence (Rebetzke et al., 2016). This results in competition for assimilates between rapidly developing awns and florets, leading to fewer fertile spikelets, reduced floret fertility, and abortion of the distal florets.

In the present study, within- and across-year analyses revealed—except for 2018, where the correlation was non-significant but positive—a positive association between AL and SA. One possible reason for the insignificant correlation in 2018 could be the extreme weather conditions. Kamal et al. (2022) showed that among the three growing seasons, highest temperature, global solar radiation, and least humidity levels were observed in 2018. The positive correlation between AL and SA, nevertheless, showed that the proportion of the aborted spikelets also increased with rapid awn growth from Waddington stage 4.5 and more. This is in line with a previously proposed hypothesis that competition between awns and floret development in the juvenile spikes might affect the number of fertile grains during harvest (Schaller and Qualset, 1975). Based on the AL ordinal scale, we also divided the whole panel into six groups and observed a general positive trend of the impact of AL on SA (**Figure S10A**). We further observed the association of geographical origin for each AL group with SA. The extent of SA varied according to the proportion of Asian, European and African accession in a given AL group, i.e., with an increase in the European and African accessions in a AL group, both AL and SA increased (**Figure S10A**). We observed that as AL increases, the grain morphometric traits and TGW also increase (**Figure S10B**), pointing towards the “source” role of the developed awns.

Interestingly, while performing multiple linear regression, AL was not one of the predictor variables for SA. Both PSN and FSN were the major predictor of SA (Kamal et al., 2022) but when one of the major predictor variables (PSN or FSN) was removed from the model, the effect of AL became significant (**Table 2**). Replacement of FSN with AL in the model leads to non-significant results for PSN but significant for AL (**Table 2A**). The non-significant results for PSN highlight that alone PSN cannot predict the variation for SA. However, FSN and AL together predict ~41% of the variation for SA (**Table 2B**) and AL alone explained ~11% of the variation in SA (**Table 2A**). Nonetheless, since our whole panel comprises only awned accessions, evaluation and comparisons of SA and AL and their effect on grain-traits in awnless or awnletted six-rowed barley would further shed light on this relationship. For example, isogenic lines of barley and wheat differing with respect to AL as well as presence and absence of awn could be useful to study their effect on SA and consequently GY (Schaller et al., 1972; Motzo and Giunta, 2002; Sanchez-Bragado et al., 2020).

**Table 2:** Multiple linear regression for spikelet abortion (SA) using potential spikelet number (PSN), final spikelet number (FSN) and awn length (AL) as predictor traits.

2a. Multiple linear regression using PSN and AL as predictor traits					
SA<- lm(SA ~ PSN + AL)					
Summary (SA)					
	Estimate	Standard error	t-value	Pr(>|t|)	Significance
(Intercept)	32.89982	2.31678	14.201	<2e-16	*** (*P* <0.001)
PSN	-0.02113	0.02218	-0.953	0.341	*ns*
AL	1.78447	0.24012	7.432	6.20E-13	*** (*P* <0.001)
Residual standard error	5.026 on 414 degrees of freedom (DF)				
Multiple R-squared	0.1179				
**Adjusted R-squared**	**0.1136**				
F-statistic	27.67 on 2 and 414 DF				
*P*-value	5.27E-12				
**2b. Multiple linear regression using FSN and AL as predictor traits**					
SA<- lm(SA ~ FSN + AL)					
Summary (SA)					
	Estimate	Standard error	t-value	Pr(>|t|)	Significance
(Intercept)	53.31362	1.70494	31.27	<2e-16	*** (*P* <0.001)
FSN	-0.34718	0.02374	-14.624	<2e-16	*** (*P* <0.001)
AL	1.31447	0.19693	6.675	7.97E-11	*** (*P* <0.001)
Residual standard error	4.086 on 414 degrees of freedom (DF)				
Multiple R-squared	0.4171				
**Adjusted R-squared**	**0.4143**				
F-statistic	148.1 on 2 and 414 DF				
*P*-value	<2.22-16				

*** and ns represent significance level at 0.001 probability (P) level and insignificant differences, respectively. The bold numbers explained the amount of variation explained by the predictor traits for SA.

### Genetic interactions of spikelet abortion with other spike- and grain-traits is generally better revealed by generalized additive models as compared to linear and quadratic regressions

In the generalized additive model (GAM), the linear predictors predict some unknown smooth monotonic function (*s*) of the expected value of the response where the response has a known mean-variance relationship (Wood et al., 2016; Hastie, 2017). Bera et al. (2021) used linear regression, polynomial regression, and GAM to study the canopy cover estimation in the dry deciduous forest of West Bengal, where they reported that GAM performed better than the other two regression models. Recently, GAM was used to predict soybean maturity under African environments (Marcillo et al., 2021). Here, we implemented linear, quadratic and GAM models to study the relationship between SA and all other 16 traits (**Figures S11**–**13**). SA was used as a response trait whose variation was explained by the individual predictor trait.

Our ANOVA to examine the model efficiency revealed that GAM outperformed both linear and quadratic regression for most of the spike traits, namely, PSN, SL, SD, and AL (**Table S5**; **Figure S11**). For FSN, however, no significant differences were observed—linear model best described the relationship between FSN and SA (**Figure S11B**). For SW, both linear and quadratic models predicted the same but higher variation in SA than GAM. Hence, either of them can be used to explain the relationship between SA and SW (**Figure S11D**).

For the grain-traits, namely, GNS, GL, GA and GS, GAM performed better than linear and quadratic regressions (**Table S5** and **Figure S12**). For GWe, however, no significant differences were observed among the three models. For GWi and TGW, we observed significant differences only between linear and GAM, where GAM explained higher variance for SA than linear regression. These results point to the complex genetics of traits per se and their complex interaction. We show that since two traits are not always related to each other linearly or quadratically, it is essential to explore the other non-linear relationship between the traits in-depth. Nevertheless, a multiple-year, multiple-location large data set gathered from a robust breeding design is essential to determine the true genetic interactions among traits.

### Increased days to heading increases PSN and FSN but decreases grain set and thousand-grain weight

Previously it was shown that HD affects spikelet initiation and growth phases in barley and wheat (Gol et al., 2017; Mulki et al., 2018; Ochagavia et al., 2018; Prieto et al., 2018; Prieto et al., 2020). Our analyses revealed that all three shoot traits, viz., HD, PH, and CDW, showed a significant positive correlation with PSN and FSN. HD showed a slightly higher correlation with PSN (*r* = 0.65; *P*<0.001) than FSN (*r* = 0.61; *P*<0.001), suggesting that with an increase in HD, the length of the spikelet initiation phase might also increase, resulting in more spikelet primordia on the spike. With an increase in PSN, the chances of retaining more spikelets (FSN) also increases. Our results showed that PSN, FSN, and HD negatively impact grain morphometric traits. Also, variation in the PC_1_ was mainly due to grain traits, and variation for PC_2_ (25.1%) could be ascribed to spike and shoot traits (**Figures 3B**, **S9A, B**); thus, confirming correlation results.

From the geographical distribution analyses, we observed that Asian accessions had lower SA, thus affecting all the grain morphometric traits. For example, in Asian accessions, we observed decreased GL, GWi, GA, GWe and TGW (**Figures 5**, **6**). In contrast, European accessions exhibited later HD, leading to higher PSN and ultimately higher SA. Since SA was higher compared to Asian accessions, this led to an increased GL, GWi, GA, GWe and ultimately TGW (**Figures 5**–**7**). Since one primary objective of any breeding program is to increase the GY by increasing the GNS or increasing the grain number per unit area while keeping grain size within a certain acceptable range, both European and Asian accessions can be exploited in the breeding schemes. The mining of favorable alleles for lower SA from Asian accessions and alleles for larger grain size and TGW from European accessions may aid in developing ideotypic spike and grain architecture, thereby altering the harvestable GY.

## Data availability statement

The raw data supporting the conclusions of this article will be made available by the authors, without undue reservation.

## Author contributions

TS: conceptualization, funding and supervision; RK: gathering the phenotypes, data analysis, and interpretation of the results; RK: writing with revisions from TS and QM; QHM: supervision of the data analyses. All authors have seen and agreed upon the final version of the manuscript.

## Funding

For conducting this study TS received financial support from the European Research Council (ERC), grant agreement 681686 ‘LUSH SPIKE,’ ERC-2015-CoG; the HEISENBERG Program of the German Research Foundation (DFG), grant no SCHN 768/8-1 and SCHN 768/15-1; and the IPK core budget. Costs for open access publishing were partially funded by the Deutsche Forschungsgemeinschaft (DFG, German Research Foundation, grant 491250510).

## Acknowledgments

We thank all the previous and present scientific members of the Plant Architecture (PBP) group for their support in phenotypic data collection. We thank Corinna Trautewig, Angelika Püschel, Franziska Backhaus, Kerstin Wolf, Mechthild Pürschel, Mattis Schnurbusch and Ellen Weiss for their outstanding technical support during all the growing seasons. We thank Dr. Guy Golan and Dr. Yongyu Huang for their valuable comments and suggestions on previous versions of the manuscript. We gratefully acknowledge the efforts of Peter Schreiber and his team for their help in managing the field experiments. We thank Martin Mascher and Sara Milner for their assistance in the barley panel selection.

## Conflict of interest

Author QHM is employed by BASF Agricultural Solutions GmbH.

The remaining authors declare that the research was conducted in the absence of any commercial or financial relationships that could be construed as a potential conflict of interest.

## Publisher’s note

All claims expressed in this article are solely those of the authors and do not necessarily represent those of their affiliated organizations, or those of the publisher, the editors and the reviewers. Any product that may be evaluated in this article, or claim that may be made by its manufacturer, is not guaranteed or endorsed by the publisher.

## Abbreviations

AL, awn length; CDW, culm dry weight; FSN, final spikelet number; GA, grain area; GAM, generalized additive model; GL, grain length; GNS, grain number per spike; GS, grain set; GWe, grain weight per spike; GWi, grain width; GY, grain yield**;** HD, heading date**;** MYP, maximum yield potential**;** PH, plant height**;** PSN, potential spikelet number; SA, spikelet abortion**;** SD, spike density; SL, spike length; SW, spike weight; TGW, thousand-grain weight.

## References

[B1] Ali M. Hussain M. Khan M. Ali Z. Zulkiffal M. Anwar J. . (2010). Source-sink relationship between photosynthetic organs and grain yield attributes during grain filling stage in spring wheat (Triticum aestivum). Int. J. Agric. Biol. 12, 509–515.

[B2] Alqudah A. M. Schnurbusch T. (2014). Awn primordium to tipping is the most decisive developmental phase for spikelet survival in barley. Funct. Plant Biol. 41, 424–436. doi: 10.1071/FP13248 32481002

[B3] Anderson P. Oelke E. Simmons S. (1995). Growth and development guide for spring barley (St. Paul, MN: University of Minnesota Extension Service).

[B4] Arisnabarreta S. Miralles D. J. (2004). The influence of fertiliser nitrogen application on development and number of reproductive primordia in field-grown two- and six-rowed barleys. Aust. J. Agric. Res. 55, 357–366. doi: 10.1071/AR03066

[B5] Bancal P. (2008). Positive contribution of stem growth to grain number per spike in wheat. Field Crops Res. 105, 27–39. doi: 10.1016/j.fcr.2007.06.008

[B6] Bancal P. (2009). Early development and enlargement of wheat floret primordia suggest a role of partitioning within spike to grain set. Field Crops Res. 110, 44–53. doi: 10.1016/j.fcr.2008.06.014

[B7] Bates D. Mächler M. Bolker B. Walker S. (2015). Fitting linear mixed-effects models using lme4. J. Stat. Software. 67, 1–48. doi: 10.18637/jss.v067.i01

[B8] Bera D. Chatterjee N. D. Bera S. (2021). Comparative performance of linear regression, polynomial regression and generalized additive model for canopy cover estimation in the dry deciduous forest of West Bengal. Remote Sens. Applications.: Soc. Environ. 22, 100502. doi: 10.1016/j.rsase.2021.100502

[B9] Bobbitt Z. (2020) How to perform quadratic regression in r. Available at: https://www.statology.org/quadratic-regression-r/ (Accessed 29.08.2022).

[B10] Cottrell J. E. Easton R. H. Dale J. E. Wadworth A. C. Adam J. S. Child R. D. . (1985). A comparison of spike and spikelet survival in mainstem and tillers of barley. Ann. Appl. Biol. 106, 365–377. doi: 10.1111/j.1744-7348.1985.tb03126.x

[B11] del Moral L. F. G. A. Del Moral M. B. G. A. Molina-Cano J. L. Slafer G. A. (2003). Yield stability and development in two-and six-rowed winter barleys under Mediterranean conditions. Field Crops Res. 81, 109–119. doi: 10.1016/S0378-4290(02)00215-0

[B12] Ellis R. Kirby E. (1980). A comparison of spring barley grown in England and in scotland. 2. yield and its components. J. Agric. Sci. 95, 111–115. doi: 10.1017/S0021859600029336

[B13] Ferrante A. Savin R. Slafer G. A. (2013). Is floret primordia death triggered by floret development in durum wheat? J. Exp. Bot. 64, 2859–2869. doi: 10.1093/jxb/ert129 23669574 PMC3741689

[B14] Garcıa del Moral L. Miralles D. Slafer G. (2002). Initiation and appearance of vegetative and reproductive structures throughout barley development. Barley. Science.: Recent Adv. Mol. Biol. to. Agron. yield. Qual., 2533–2335.

[B15] Gol L. Tome F. Von Korff M. (2017). Floral transitions in wheat and barley: Interactions between photoperiod, abiotic stresses, and nutrient status. J. Exp. Bot. 68, 1399–1410. doi: 10.1093/jxb/erx055 28431134

[B16] Gonzalez F. G. Miralles D. J. Slafer G. A. (2011). Wheat floret survival as related to pre-anthesis spike growth. J. Exp. Bot. 62, 4889–4901. doi: 10.1093/jxb/err182 21705386

[B17] González-Navarro O. E. Griffiths S. Molero G. Reynolds M. P. Slafer G. A. (2015). Dynamics of floret development determining differences in spike fertility in an elite population of wheat. Field Crops Res. 172, 21–31. doi: 10.1016/j.fcr.2014.12.001

[B18] González F. G. Slafer G. A. Miralles D. J. (2003). Floret development and spike growth as affected by photoperiod during stem elongation in wheat. Field Crops Res. 81, 29–38. doi: 10.1016/S0378-4290(02)00196-X

[B19] Guo Z. Schnurbusch T. (2016). Costs and benefits of awns. J. Exp. Bot. 67(9), 2533–2535. doi: 10.1093/jxb/erw140 27162273 PMC4861032

[B20] Hastie T. J. (2017). Generalized additive models. In Statistical models in S. (pp. 249–307) (Routledge).

[B21] Kamal R. Muqaddasi Q. H. Zhao Y. Schnurbusch T. (2022). Spikelet abortion in six-rowed barley is mainly influenced by final spikelet number, with potential spikelet number acting as a suppressor trait. J. Exp. Bot., 73 (7), 2005–2020. doi: 10.1093/jxb/erab529 34864992

[B22] Kirby E. (1988). Analysis of leaf, stem and ear growth in wheat from terminal spikelet stage to anthesis. Field Crops Res. 18, 127–140. doi: 10.1016/0378-4290(88)90004-4

[B23] Kirby E. Appleyard M. (1987). “Development and structure of the wheat plant,” in Wheat breeding (Dordrecht: Springer), 287–311.

[B24] Knott D. R. (1986). Effect of genes for photoperiodism, semidwarfism, and awns on agronomic characters in a wheat cross. Crop Sci. 26, 1158–1162. doi: 10.2135/cropsci1986.0011183X002600060016x

[B25] Komatsuda T. Pourkheirandish M. He C. Azhaguvel P. Kanamori H. Perovic D. . (2007). Six-rowed barley originated from a mutation in a homeodomain-leucine zipper I-class homeobox gene. Proc. Natl. Acad. Sci. 104, 1424–1429. doi: 10.1073/pnas.0608580104 17220272 PMC1783110

[B26] Lê S. Josse J. Husson F. (2008). FactoMineR: An R package for multivariate analysis. J. Stat. software. 25, 1–18. doi: 10.18637/jss.v025.i01

[B27] Li X. Wang H. Li H. Zhang L. Teng N. Lin Q. . (2006). Awns play a dominant role in carbohydrate production during the grain-filling stages in wheat (Triticum aestivum). Physiol. plant. 127, 701–709. doi: 10.1111/j.1399-3054.2006.00679.x

[B28] Marcillo G. S. Martin N. F. Diers B. W. Da Fonseca Santos M. Leles E. P. Chigeza G. . (2021). Implementation of a generalized additive model (GAM) for soybean maturity prediction in African environments. Agronomy 11, 1043. doi: 10.3390/agronomy11061043

[B29] Martin J. N. Carver B. F. Hunger R. M. Cox T. S. (2003). Contributions of leaf rust resistance and awns to agronomic and grain quality performance in winter wheat. Crop science, 43 (5), 1712–1717 doi: 10.2135/cropsci2003.1712

[B30] McKenzie H. (1972). Adverse influence of awns on yield of wheat. Can. J. Plant Sci. 52, 81–87. doi: 10.4141/cjps72-010

[B31] Miralles D. J. Katz S. D. Colloca A. Slafer G. A. (1998). Floret development in near isogenic wheat lines differing in plant height. Field Crops Res. 59, 21–30. doi: 10.1016/S0378-4290(98)00103-8

[B32] Miralles D. J. Richards R. A. (2000). Responses of leaf and tiller emergence and primordium initiation in wheat and barley to interchanged photoperiod. Ann. Bot. 85, 655–663. doi: 10.1006/anbo.2000.1121

[B33] Motzo R. Giunta F. (2002). Awnedness affects grain yield and kernel weight in near-isogenic lines of durum wheat. Aust. J. Agric. Res. 53, 1285–1293. doi: 10.1071/AR02008

[B34] Mulki M. A. Bi X. Von Korff M. (2018). FLOWERING LOCUS T3 controls spikelet initiation but not floral development. Plant Physiol. 178, 1170–1186. doi: 10.1104/pp.18.00236 30213796 PMC6236595

[B35] Muqaddasi Q. H. Brassac J. Ebmeyer E. Kollers S. Korzun V. Argillier O. . (2020). Prospects of GWAS and predictive breeding for european winter wheat’s grain protein content, grain starch content, and grain hardness. Sci. Rep. 10, 1–17. doi: 10.1038/s41598-020-69381-5 32719416 PMC7385145

[B36] Ochagavia H. Prieto P. Savin R. Griffiths S. Slafer G. (2018). Dynamics of leaf and spikelet primordia initiation in wheat as affected by ppd-1a alleles under field conditions. J. Exp. Bot. 69, 2621–2631. doi: 10.1093/jxb/ery104 29562296 PMC5920321

[B37] Prieto P. Ochagavía H. Griffiths S. Slafer G. A. (2020). Earliness per se× temperature interaction: Consequences on leaf, spikelet, and floret development in wheat. J. Exp. Bot. 71, 1956–1968. doi: 10.1093/jxb/erz568 31875911 PMC7242086

[B38] Prieto P. Ochagavía H. Savin R. Griffiths S. Slafer G. A. (2018). Dynamics of floret initiation/death determining spike fertility in wheat as affected by ppd genes under field conditions. J. Exp. Bot. 69, 2633–2645. doi: 10.1093/jxb/ery105 29562264 PMC5920323

[B39] Rebetzke G. Bonnett D. Reynolds M. (2016). Awns reduce grain number to increase grain size and harvestable yield in irrigated and rainfed spring wheat. J. Exp. Bot. 67, 2573–2586. doi: 10.1093/jxb/erw081 26976817 PMC4861010

[B40] Reynolds M. Foulkes J. Furbank R. Griffiths S. King J. Murchie E. . (2012). Achieving yield gains in wheat. Plant. Cell Environ. 35, 1799–1823. doi: 10.1111/j.1365-3040.2012.02588.x 22860982

[B41] Sanchez-Bragado R. Kim J. Rivera-Amado C. Molero G. Araus J. Savin R. . (2020). Are awns truly relevant for wheat yields? a study of performance of awned/awnless isogenic lines and their response to source–sink manipulations. Field Crops Res. 254, 107827. doi: 10.1016/j.fcr.2020.107827

[B42] Schaller C. Qualset C. (1975). Isogenic analysis of productivity in barley: Interaction of genes affecting awn length and leaf-spotting. Crop Sci. 15, 378–382. doi: 10.2135/cropsci1975.0011183X001500030029x

[B43] Schaller C. Qualset C. O. Rutger J. N. (1972). Isogenic analysis of the effects of the awn on productivity of barley. Crop Sci. 12, 531–535. doi: 10.2135/cropsci1972.0011183X001200040046x

[B44] Su X. Yan X. Tsai C. L. (2012). Linear regression. Wiley. Interdiscip. Reviews.: Comput. Stat 4, 275–294. doi: 10.1002/wics.1198

[B45] Team, R.C (2013). R: A language and environment for statistical computing. (Vienna, Austria: R Foundation for statistical computing)

[B46] Thirulogachandar V. Koppolu R. Schnurbusch T. (2021). Strategies of grain number determination differentiate barley row types. J. Exp. Bot. 72 (22), 7754–7768. doi: 10.1093/jxb/erab395 34460900

[B47] Thirulogachandar V. Schnurbusch T. (2021). ‘Spikelet stop’ determines the maximum yield potential stage in barley. J. Exp. Bot., erab342. doi: 10.1093/jxb/erab1342 PMC8643653 34291795

[B48] Vervelde G. (1953). The agricultural value of awns in cereals. Netherlands J. Agric. Sci. 1, 2–10. doi: 10.18174/njas.v1i1.17881

[B49] Weyhrich R. A. Carver B. F. Smith E. L. (1994). Effect of awn suppression on grain yield and agronomic traits in hard red winter wheat. Crop Sci. 34, 965–969. doi: 10.2135/cropsci1994.0011183X003400040025x

[B50] Wood S. N. (2006). Generalized additive models: An introduction with R (New York: chapman and hall/CRC).

[B51] Wood S. N. Pya N. Säfken B. (2016). Smoothing parameter and model selection for general smooth models. J. Am. Stat. Assoc. 111, 1548–1563. doi: 10.1080/01621459.2016.1180986

[B52] Wood S. Wood M. S. (2015). Package ‘mgcv’. R package version. 1 (29), 729

[B53] Zhang Z. Li J. Hu N. Li W. Qin W. Li J. . (2021). Spike growth affects spike fertility through the number of florets with green anthers before floret abortion in wheat. Field Crops Res. 260, 108007. doi: 10.1016/j.fcr.2020.108007

[B54] Zhu Y. Chu J. Dai X. He M. (2019). Delayed sowing increases grain number by enhancing spike competition capacity for assimilates in winter wheat. Eur. J. Agron. 104, 49–62. doi: 10.1016/j.eja.2019.01.006

