# Supplementary material for: Genetic association of spikelet abortion with spike, grain, and shoot traits in highly-diverse six-rowed barley
Source: Front Plant Sci. 2022 Nov 21;13:1015609. doi: 10.3389/fpls.2022.1015609 (PMC9719993; doi:10.3389/fpls.2022.1015609)
Supplement: Supplementary file 1 [file DataSheet_1.pdf]

Supplementary Figures

Article type: **Research article**

Genetic association of spikelet abortion with spike, grain, and shoot traits in highly-diverse six-rowed barley

Roop Kamal [1,*], Quddoos H. Muqaddasi [1,†], and Thorsten Schnurbusch [1, 2,*]

[1] Leibniz Institute of Plant Genetics and Crop Plant Research (IPK), Gatersleben, Germany
[2] Faculty of Natural Sciences III, Institute of Agricultural and Nutritional Sciences, Martin Luther University Halle-Wittenberg, Halle, Germany
[†] Present address: European Wheat Breeding Center, BASF Agricultural Solutions GmbH, Gatersleben, Germany

* Corresponding authors: kamal@ipk-gatersleben.de; schnurbusch@ipk-gatersleben.de

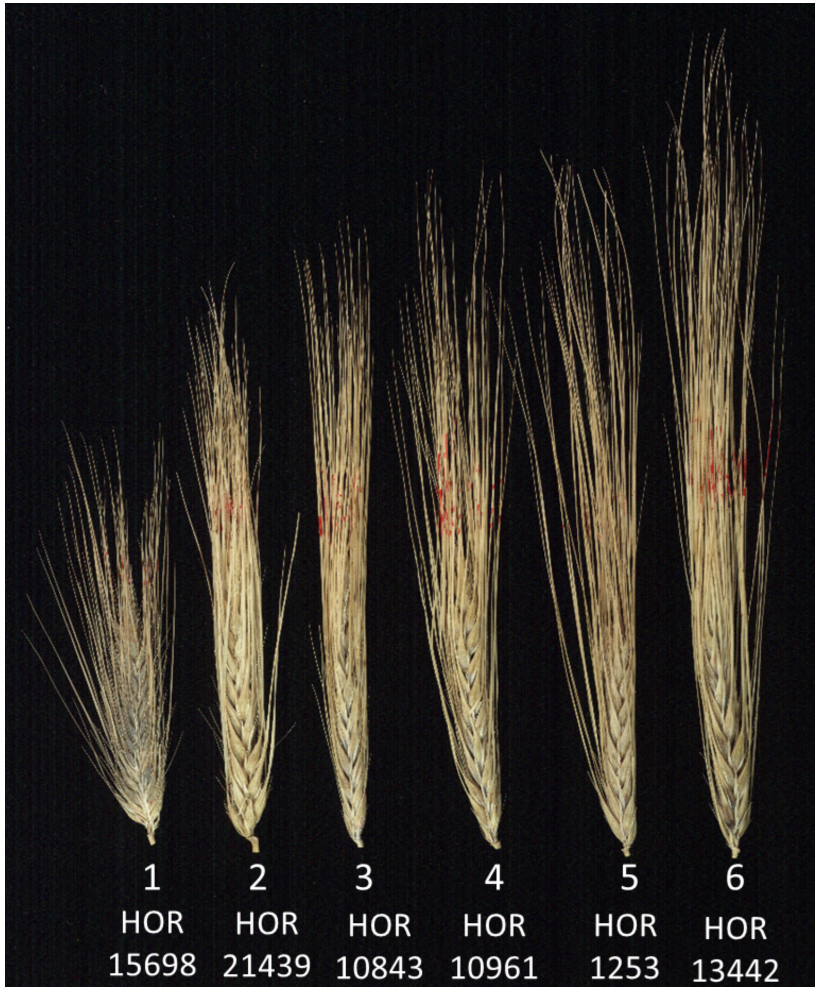

Figure S1: Representative samples for awn length in six-rowed barley panel. The awn length range from one to six. The accession names for the respective awn length number are mentioned in the image.

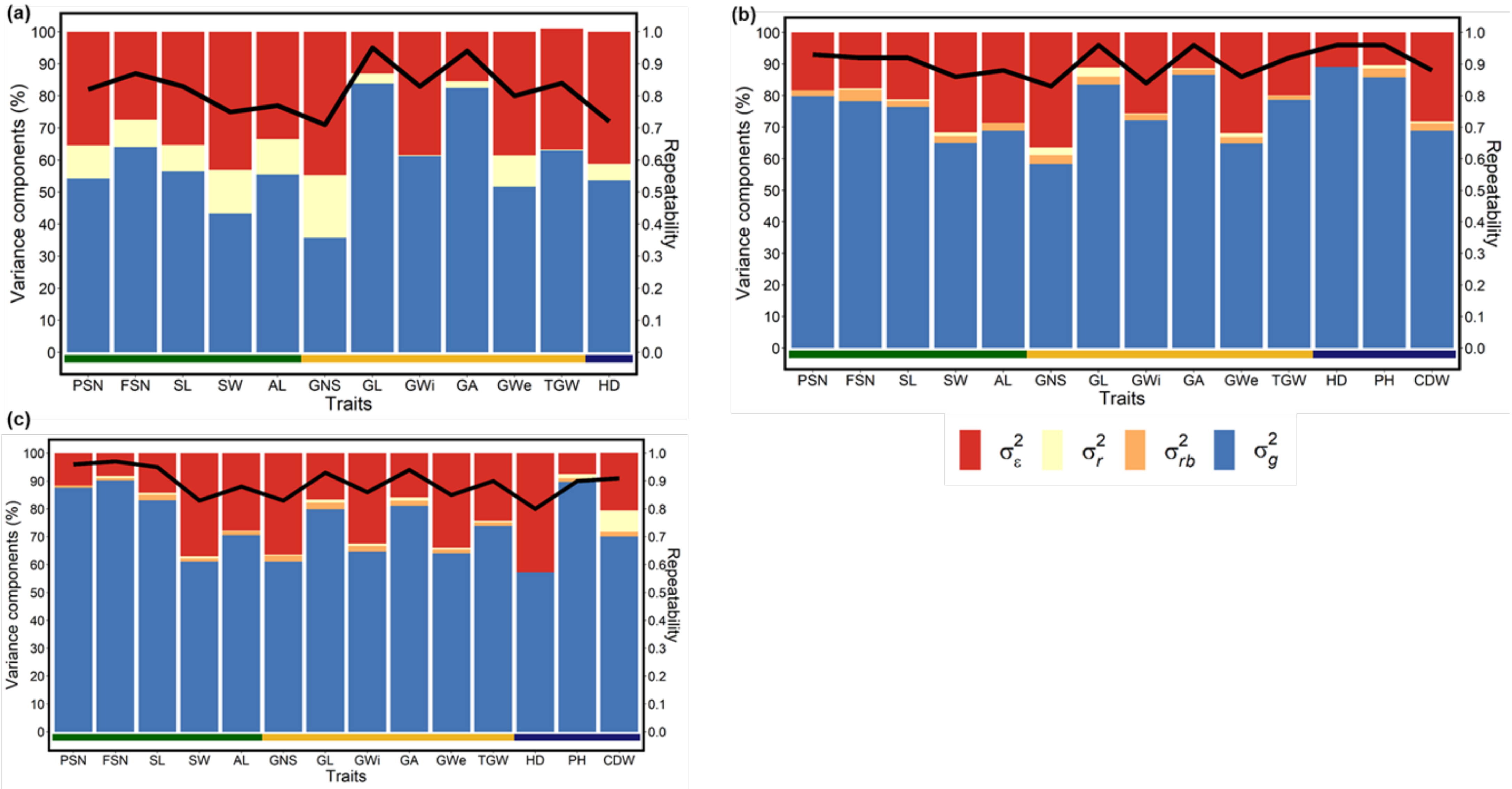

Figure S2: Proportion of the different variance components and repeatability for each investigated trait. (a) Variance component and repeatability in 2018. (b) Variance component and repeatability in 2019. (c) Variance component and repeatability in 2020. The *x*-axis represents all investigated traits, the left *y*-axis denotes the proportion of the variance components in percent, and the right *y*-axis represents the repeatability scores. The black line represents the repeatability value for the respective trait, σ_g^2 is the genotypic variance, σ_r^2 is the replication variance, σ_{rb}^2 is the replication nested in blocks variance and σ_ε^2 is the error or residual variance. The spike traits indicated by the green horizontal line include potential spikelet number (PSN), final spikelet number (FSN), spike length (SL in cm), spike weight (SW in g), and awn length (AL). Grain traits represented by the yellow horizontal line include grain number per spike (GNS), grain length (GL in mm), grain width (GWi in mm), grain area (GA in mm^2), grain weight per spike (GWe in g), thousand-grain weight (TGW in g) and the shoot trait represented by blue horizontal line include heading date (HD in days from January 1st) plant height (PH in cm) and culm dry weight (CDW in g).

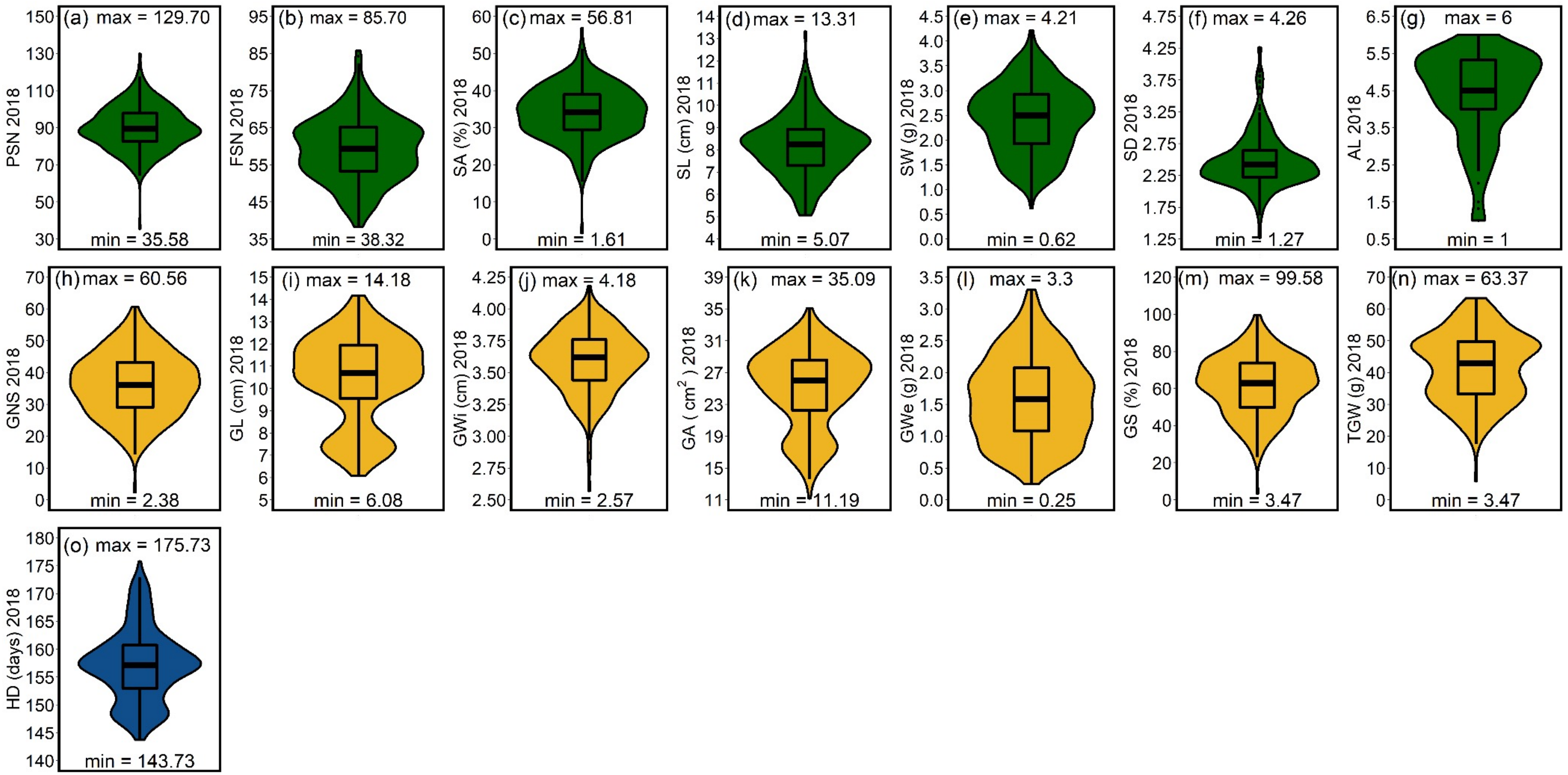

Figure S3: Phenotypic distribution of the investigated traits in a panel of 288 six-rowed spring barley accessions in 2018. (a-g) frequency distribution for the spike traits, (h-n) frequency distribution for grain traits and (o) frequency distribution for the shoot trait. "max" and "min" represents the maximum and minimum value for each investigated trait and the box plot within the violin plots represents the lower quartile, median and upper quartile for each trait. (a) potential spikelet number (PSN), (b) final spikelet number (FSN), (c) spikelet abortion (SA in %), (d) spike length (SL in cm), (e) spike weight (SW in g), (f) spike density (SD); (g) awn length (AL), (h) grain number per spike (GNS), (i) grain length (GL in mm), (j) grain width (GWi in mm), (k) grain area (GA in mm^2), (l) grain weight (GWe in g), (m) grain set (GS in %), (n) thousand-grain weight (TGW in g), (o) heading date (HD in days from January 1st), (p) plant height (PH in cm) and (q) culm dry weight (CDW in g).

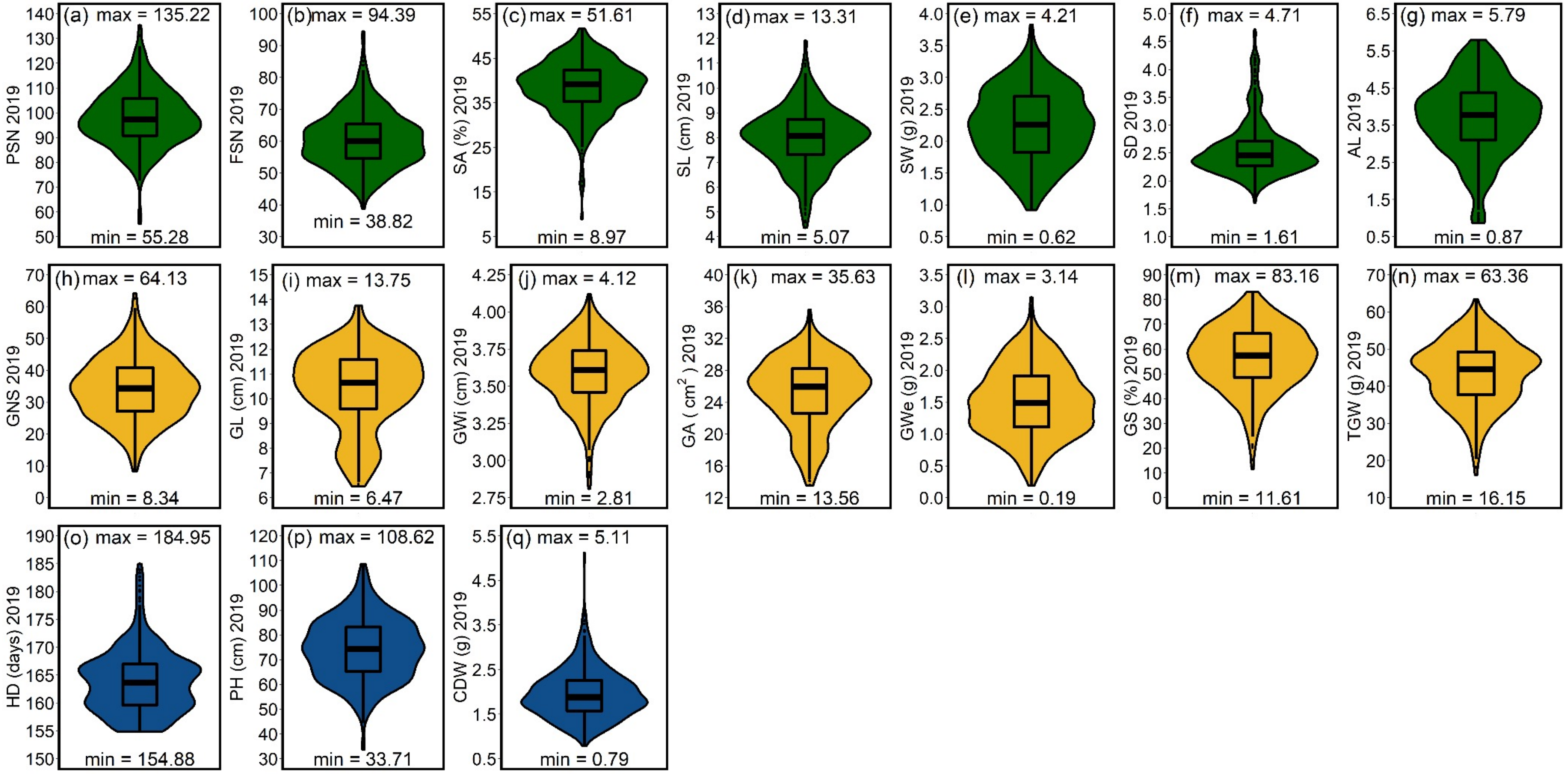

Figure S4: Phenotypic distribution of the investigated traits in a panel of 417 six-rowed spring barley accessions in 2019. (a-g) frequency distribution for the spike traits, (h-n) frequency distribution for grain traits and (o-q) frequency distribution for shoot traits . “max” and “min” represents the maximum and minimum value for each investigated trait and the box plot within the violin plots represents the lower quartile, median and upper quartile for each trait. (a) potential spikelet number (PSN), (b) final spikelet number (FSN), (c) spikelet abortion (SA in %), (d) spike length (SL in cm), (e) spike weight (SW in g), (f) spike density (SD); (g) awn length (AL), (h) grain number per spike (GNS), (i) grain length (GL in mm), (j) grain width (GWi in mm), (k) grain area (GA in mm^2), (l) grain weight (GWe in g), (m) grain set (GS in %), (n) thousand-grain weight (TGW in g), (o) heading date (HD in days from January 1st), (p) plant height (PH in cm) and (q) culm dry weight (CDW in g).

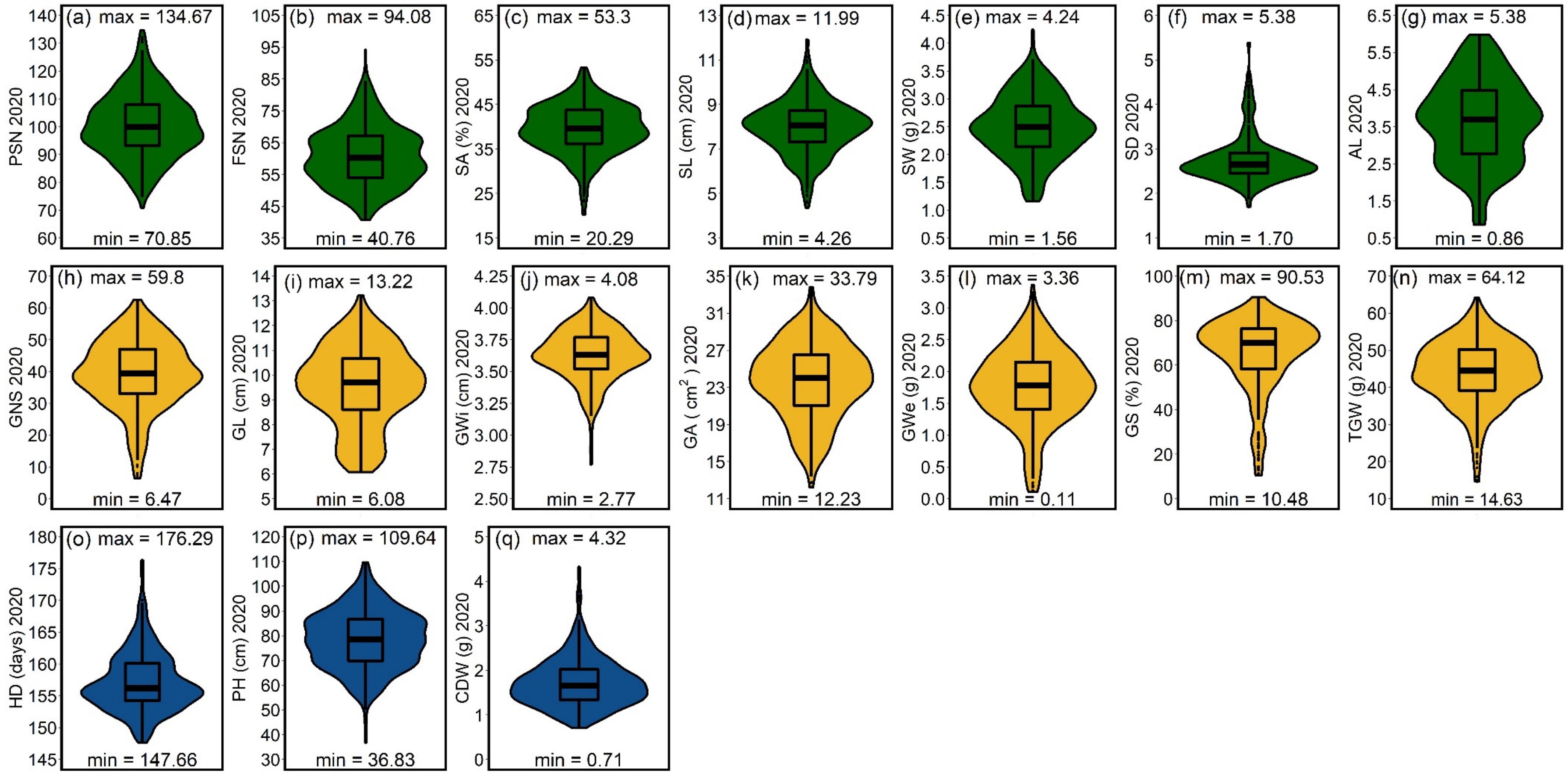

Figure S5: Phenotypic distribution of the investigated traits in a panel of 417 six-rowed spring barley accessions in 2020. (a-g) frequency distribution for the spike traits, (h-n) frequency distribution for grain traits and (o-q) frequency distribution for shoot traits. "max" and "min" represents the maximum and minimum value for each investigated trait and the box plot within the violin plots represents the lower quartile, median and upper quartile for each trait. (a) potential spikelet number (PSN), (b) final spikelet number (FSN), (c) spikelet abortion (SA in %), (d) spike length (SL in cm), (e) spike weight (SW in g), (f) spike density (SD); (g) awn length (AL), (h) grain number per spike (GNS), (i) grain length (GL in mm), (j) grain width (GWi in mm), (k) grain area (GA in mm^2), (l) grain weight (GWe in g), (m) grain set (GS in %), (n) thousand-grain weight (TGW in g), (o) heading date (HD in days from January 1st), (p) plant height (PH in cm) and (q) culm dry weight (CDW in g).

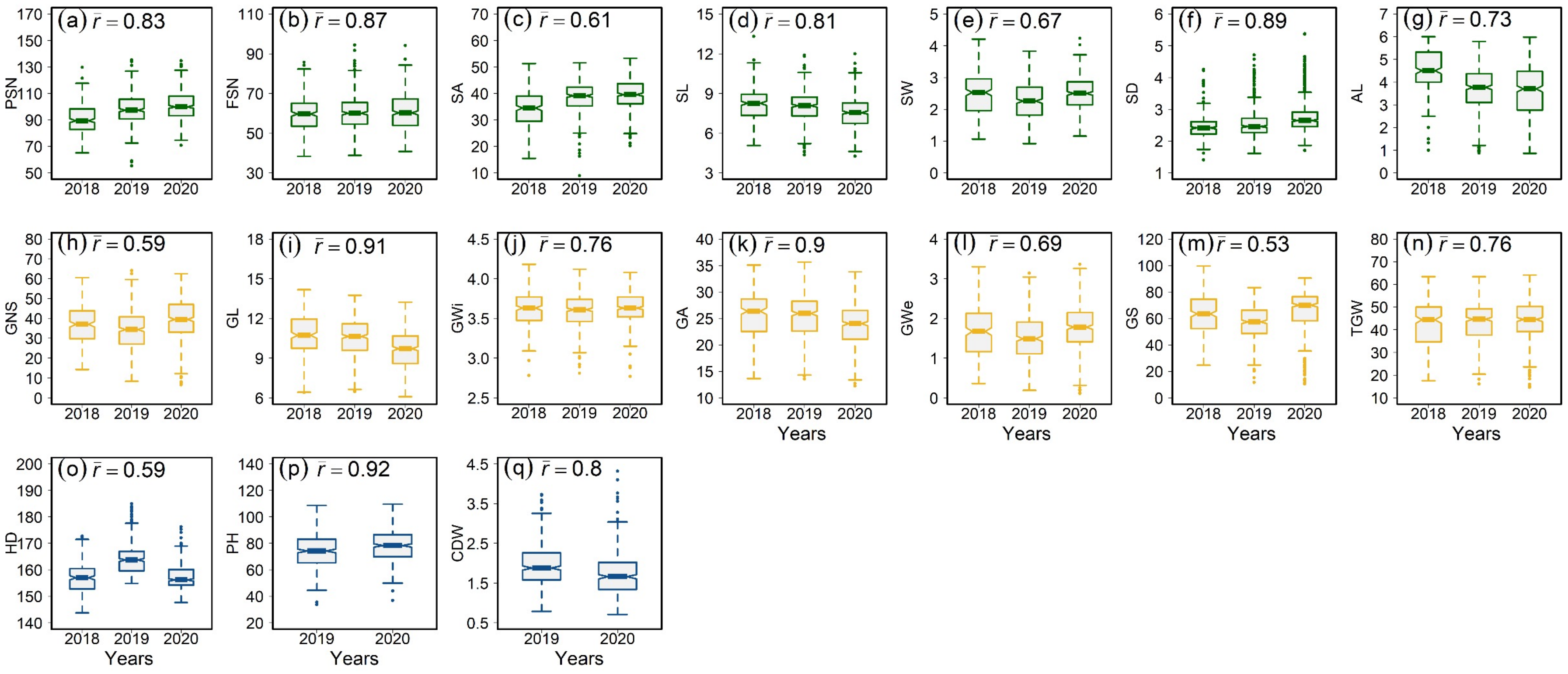

Figure S6: Environment (year) specific phenotypic distribution of the investigated traits in a panel of 417 six-rowed spring barley accessions with between years average trait correlation ($\bar{r}$) calculated by performing the Fisher's *z* transformation. (a-g) average correlation for the spike traits, (h-n) average correlation for grain traits and (o-q) average correlation for shoot traits. The *x*- and *y*-axis of each plot indicate the years and the particular studied trait, respectively. (a) potential spikelet number (PSN), (b) final spikelet number (FSN), (c) spikelet abortion (SA in %), (d) spike length (SL in cm), (e) spike weight (SW in g), (f) spike density (SD); (g) awn length (AL), (h) grain number per spike (GNS), (i) grain length (GL in mm), (j) grain width (GWi in mm), (k) grain area (GA in mm^2), (l) grain weight (GWe in g), (m) grain set (GS in %), (n) thousand-grain weight (TGW in g), (o) heading date (HD in days from January 1st), (p) plant height (PH in cm) and (q) culm dry weight (CDW in g)

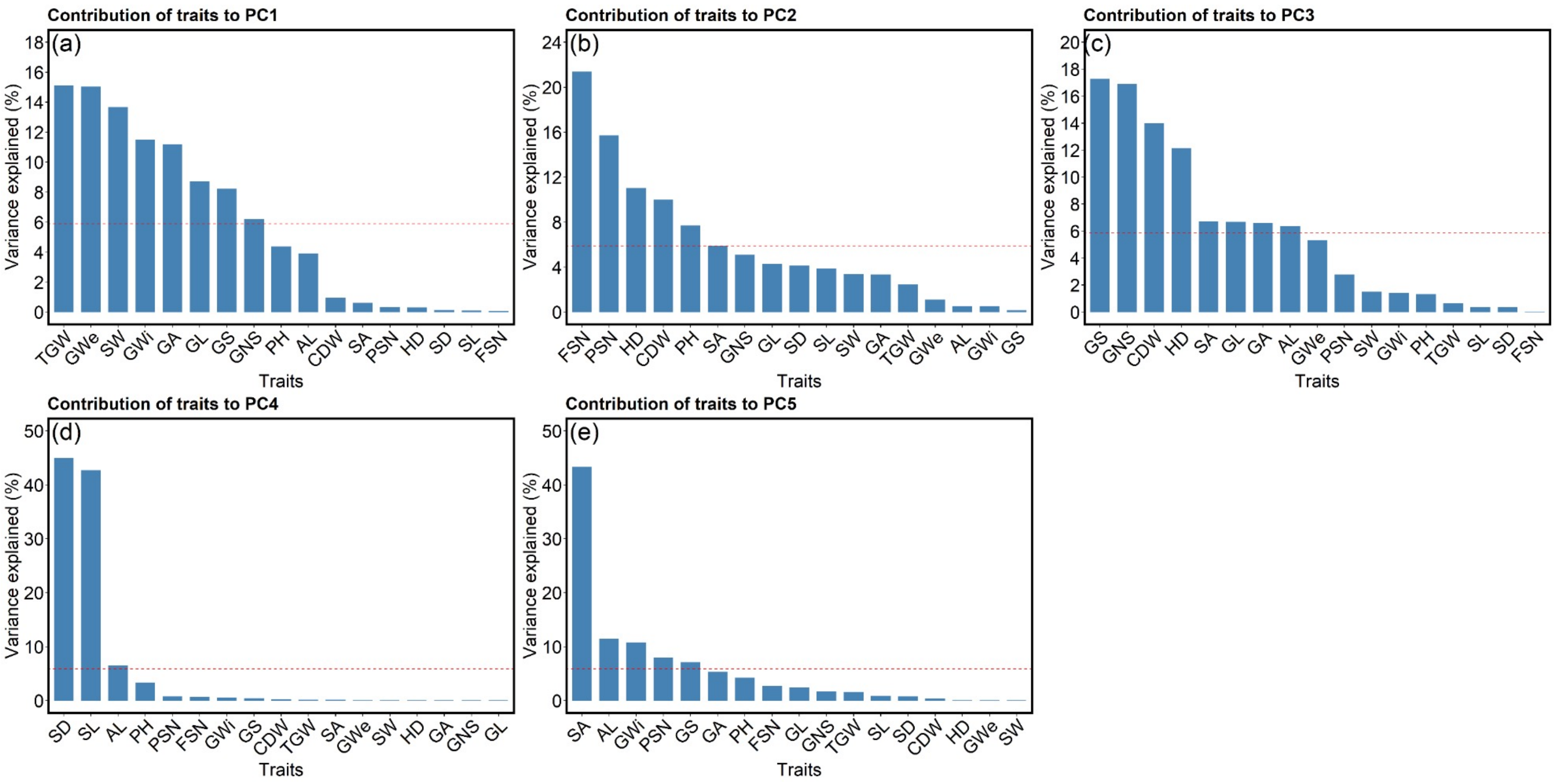

Figure S7: Contribution of the traits to the individual principal components (PCs). The *x*- and *y*-axis in each graph explains the traits and variance explained in percent, respectively. (a) Contribution of the traits to PC1, (b) contribution of the traits to PC2, (c) contribution of the traits to PC3, (d) contribution of the traits to PC4 and (e) contribution of the traits to PC5. PSN = potential spikelet number; FSN = final spikelet number; SA = spikelet abortion (SA in %); SL = spike length (in cm); SW = spike weight (in g); SD = spike density; AL = awn length; GNS = grain number per spike; GL = grain length (in mm); GWi = grain width (in mm); GA = grain area (in mm^2); GWe = grain weight (in g); GS = grain set (in %); TGW = thousand-grain weight (in g); HD = heading date (in days from January 1st); PH = plant height (in cm) and CDW = culm dry weight (in g).

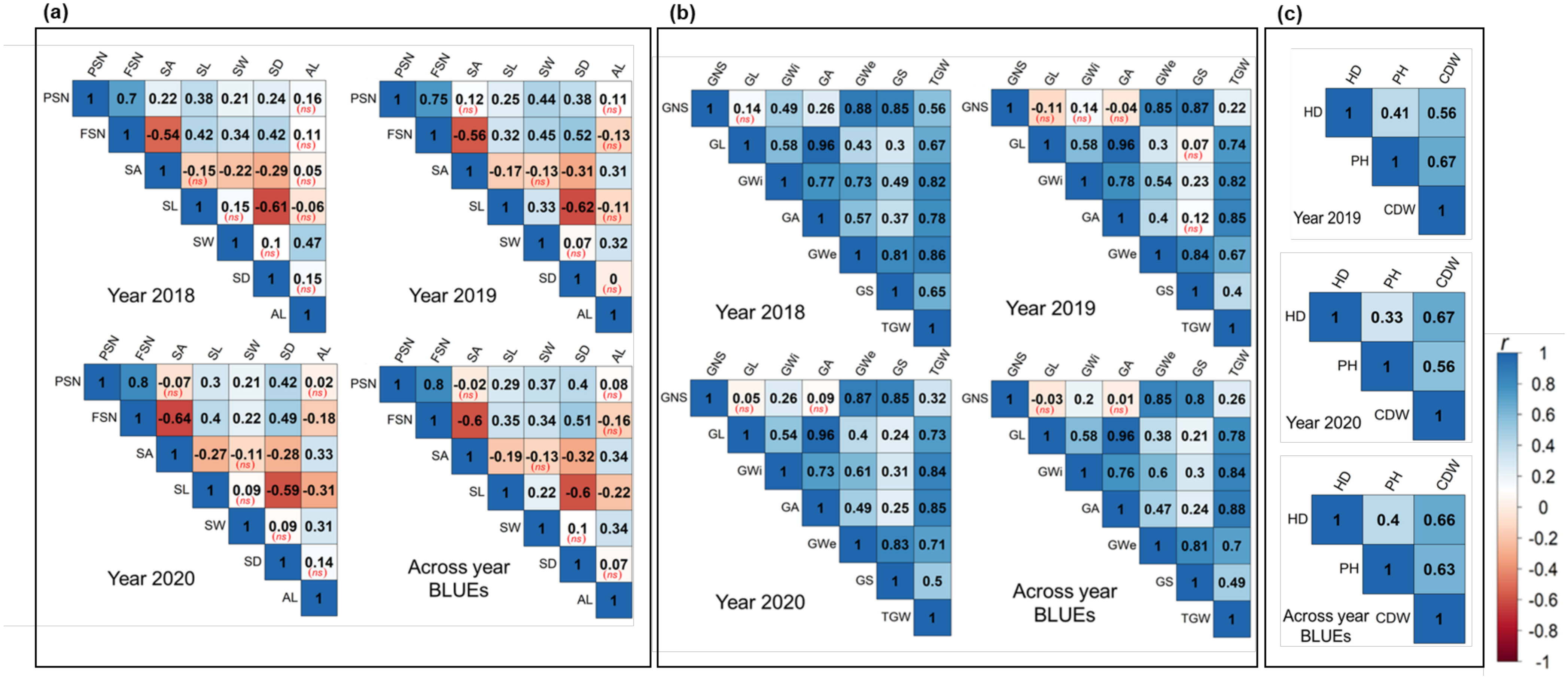

Figure S8: Within- and across-year Pearson-product moment correlation (*r*) analyses for (a) spike traits, (b) grain traits and (c) shoot traits.'*(ns)*' shows the insignificant correlation among the traits at $P < 0.001$. Spike traits include potential spikelet number (PSN), final spikelet number (FSN), spikelet abortion (SA), spike length (SL in cm), spike weight (SW in g), spike density (SD) and awn length (AL). Grain traits include grain number per spike (GNS), grain length (GL in mm), grain width (GWi in mm), grain area (GA in mm^2), grain weight (GWe in g), grain set (GS in %), and thousand-grain weight (TGW in g). Shoot traits include in heading date (HD in days from January 1st), plant height (PH in cm) and culm dry weight (CDW in g)

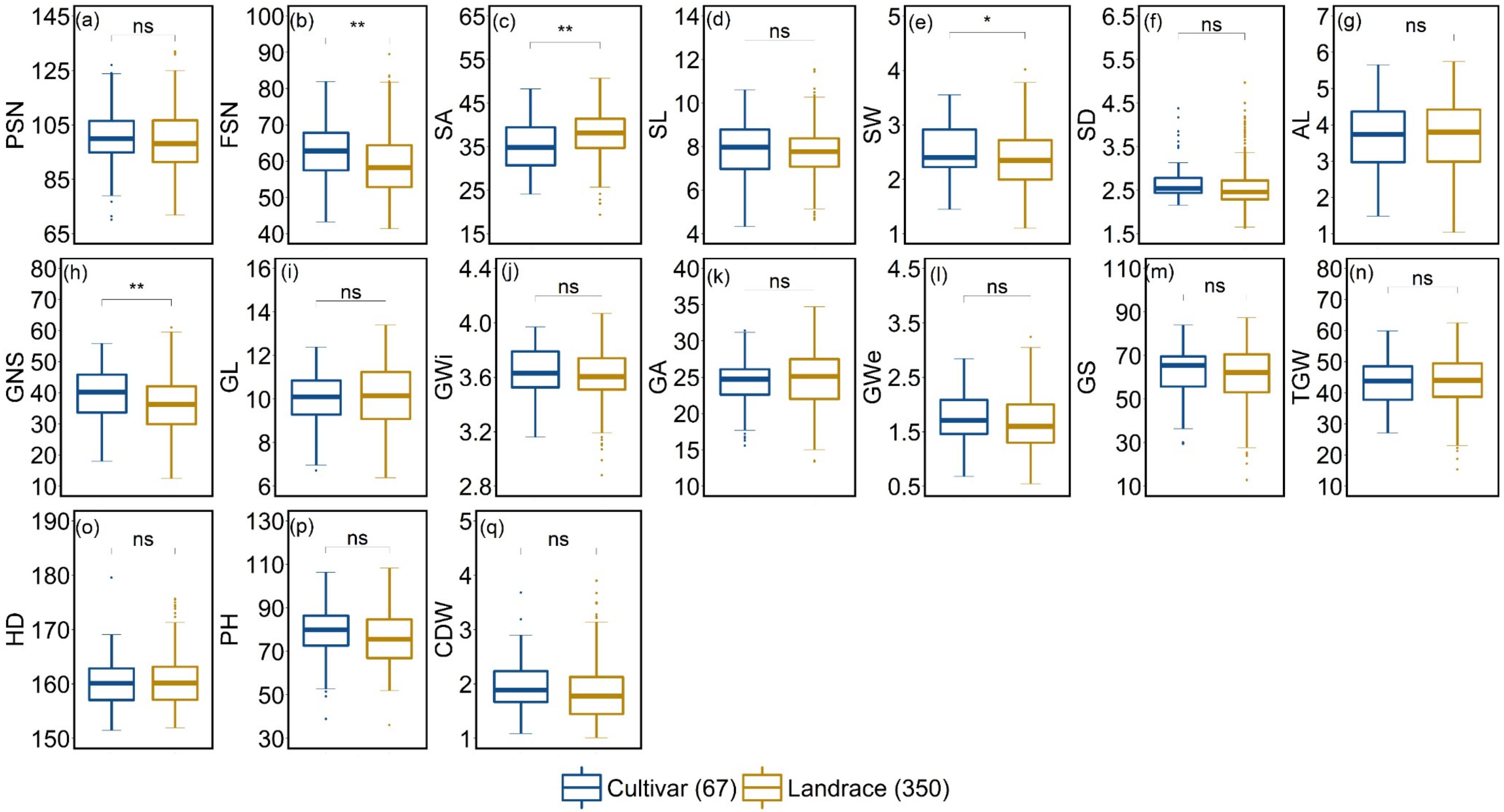

Figure S9: Comparison of accessions with respect to accession nature, viz., cultivar and landraces (a) potential spikelet number (PSN), (b) final spikelet number (FSN), (c) spikelet abortion (SA in %), (d) spike length (SL in cm), (e) spike weight (SW in g), (f) spike density (SD); (g) awn length (AL), (h) grain number per spike (GNS), (i) grain length (GL in mm), (j) grain width (GWi in mm), (k) grain area (GA in mm^2), (l) grain weight (GWe in g), (m) grain set (GS in %), (n) thousand-grain weight (TGW in g), (o) heading date (HD in days from January 1st), (p) plant height (PH in cm) and (q) culm dry weight (CDW in g). '*n*' denotes the number of accessions; **= 0.01 probability level; *= 0.05 and 'ns' represents insignificant differences.

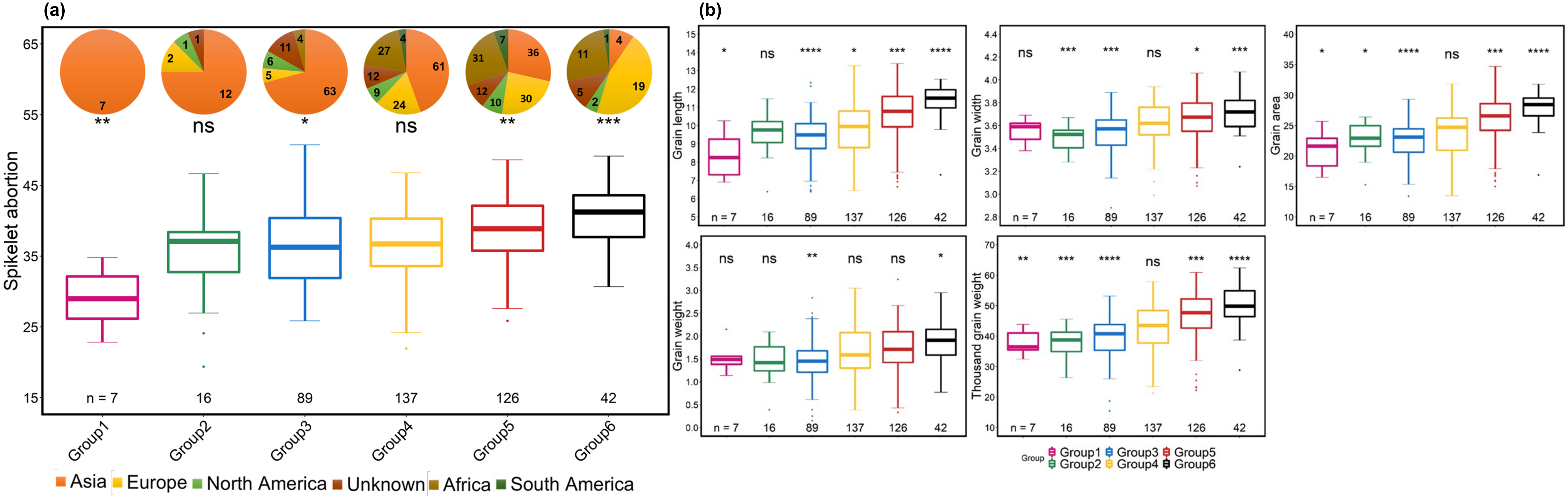

Figure S10: (a) Distribution (grouping) of the 417 barley accessions based on their awn length (AL) on 1 to 6 ordinal scale. The Pie charts within the figure represent the continent-wise distribution of AL in each group. (b) Effect of awn length (AL) on the grain traits. The accessions are grouped with AL ranging from 0−1 (group1) , 1−2 (group2) , 2−3 (group3) , 3−4 (group4) , 4−5 (group5) , and 5−6 (group6). *, **, ***, and *ns* represent the significance (*P*) values based on student's t-test as *P* <0.05, *P* <0.01, *P* <0.001, and *P* >0.5, respectively.

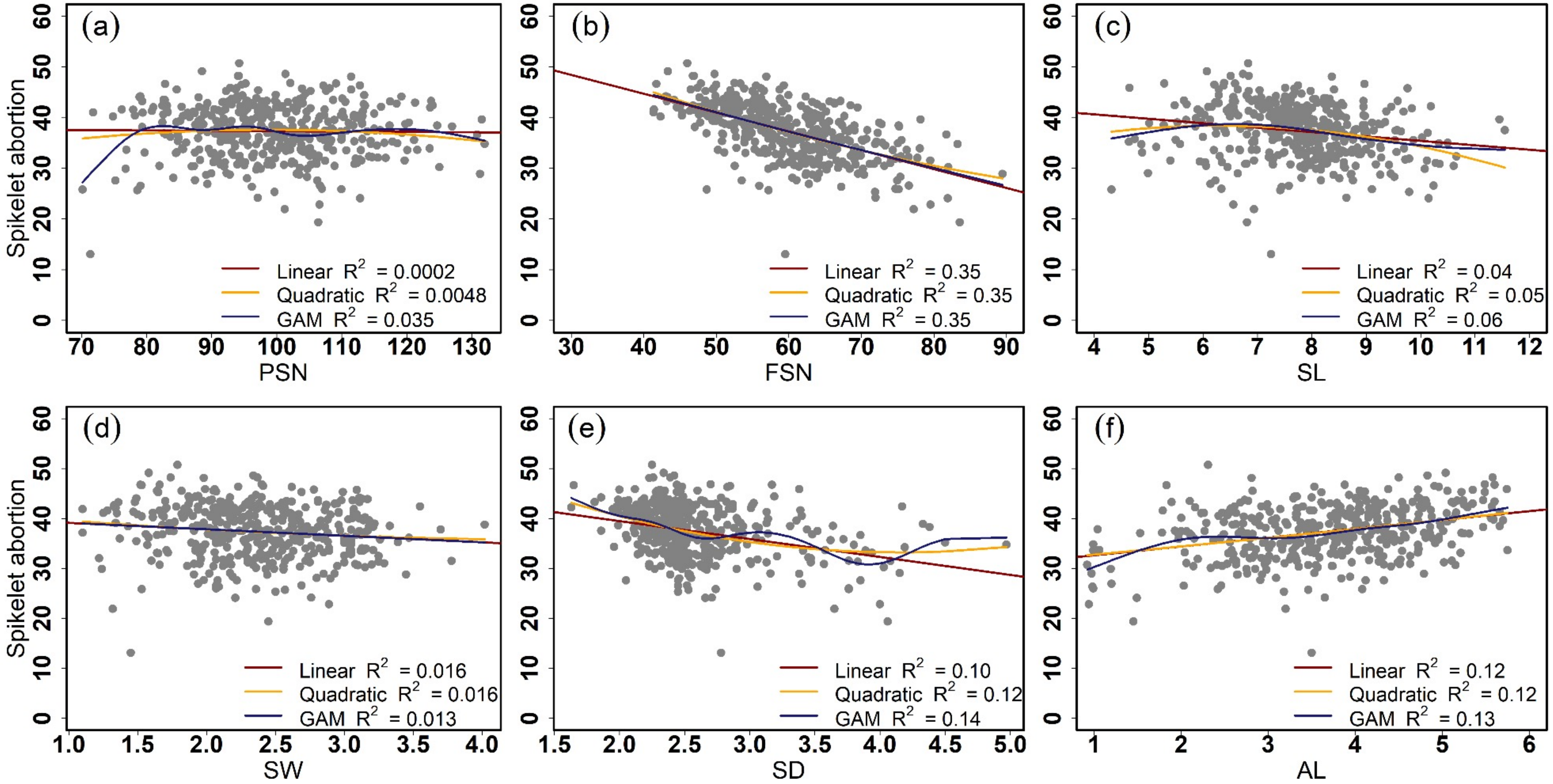

Figure S11: Comparison of different regression models explaining the relationship between spikelet abortion and all the investigated spike traits. The *x*- and *y*-axis represent the different spike traits and spikelet abortion respectively. (a) regression analysis between potential spikelet number and spikelet abortion, (b) regression analysis between final spikelet number and spikelet abortion, (c) regression analysis between spike length and spikelet abortion, (d) regression analysis between spike weight and spikelet abortion, (e) regression analysis between spike density and spikelet abortion and (f) regression analysis between awn length and spikelet abortion. The red line shows the linear regression, the yellow line indicates the quadratic regression, the blue line indicates the generalized additive model (GAM) and R^2 is the coefficient of determination.

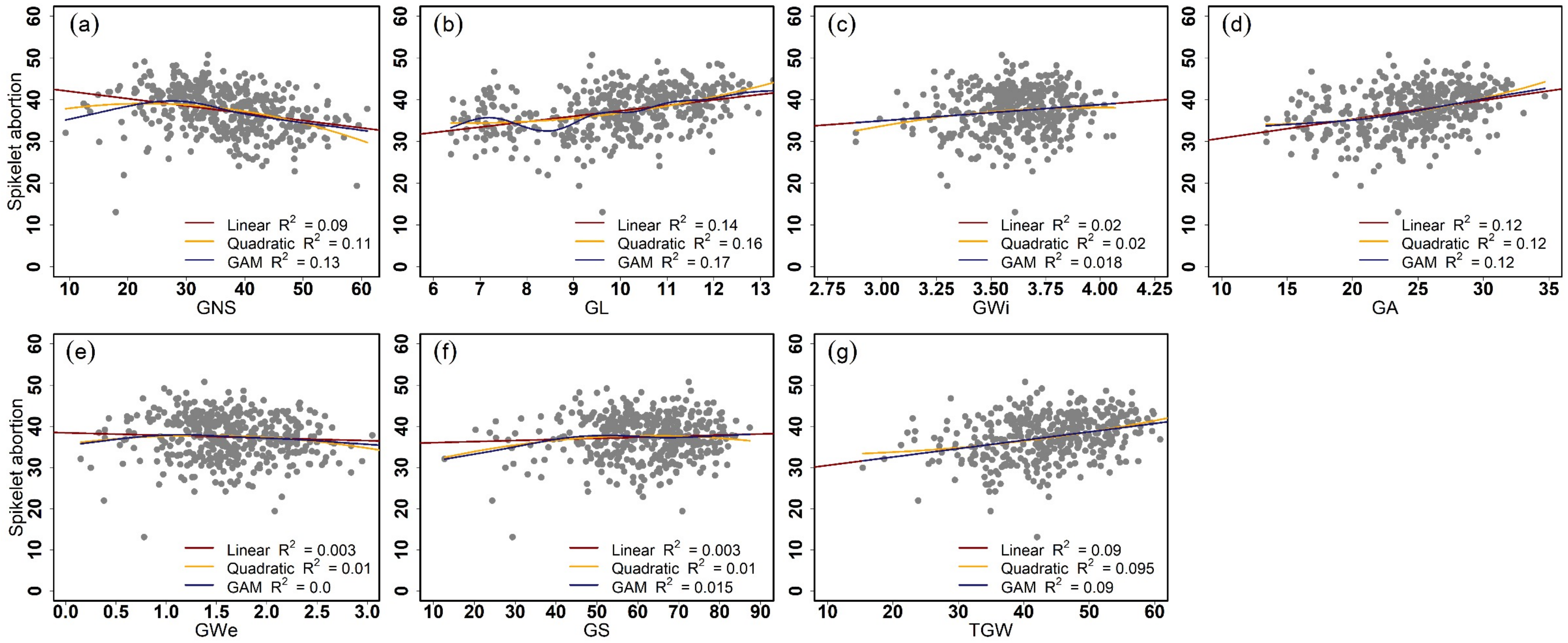

Figure S12: Comparison of different regression models explaining the relationship between spikelet abortion and all the investigated grain traits. The *x*- and *y*-axis represent the different grain traits and spikelet abortion respectively. (a) regression analysis between grain number per spike and spikelet abortion, (b) regression analysis between grain length and spikelet abortion, (c) regression analysis between grain width and spikelet abortion, (d) regression analysis between grain area and spikelet abortion, (e) regression analysis between grain weight and spikelet abortion, (f) regression analysis between grain fertility and spikelet abortion and (g) regression analysis between thousand-grain weight and spikelet abortion. The red line shows the linear regression, the yellow line indicates the quadratic regression, the blue line indicates the generalized additive model (GAM) and R^2 is the coefficient of determination.

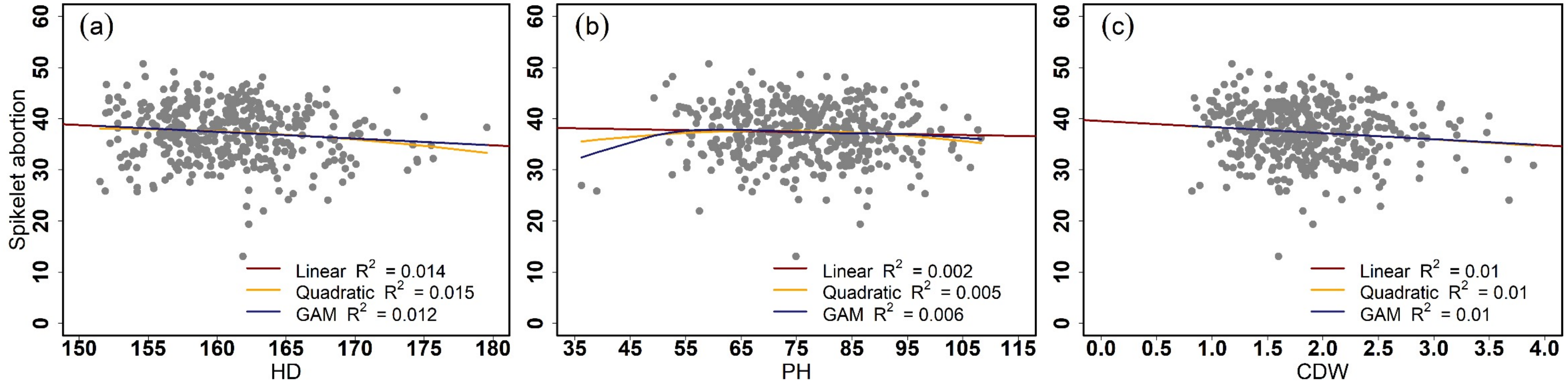

Figure S13: Comparison of different regression models explaining the relationship between spikelet abortion and all the investigated shoot traits. The *x*- and *y*- axis represents the different agronomic traits and spikelet abortion respectively. (a) regression analysis between heading date and spikelet abortion, (b) regression analysis between plant height and spikelet abortion, and (c) regression analysis between culm dry weight and spikelet abortion. The red line shows the linear regression, the yellow line indicates the quadratic regression, the blue line indicates the generalized additive model (GAM) and R^2 is the coefficient of determination.
